# Defect-induced ultimately fast volume phonon-polaritons in the wurtzite Zn_0.74_Mg_0.26_Se mixed crystal

**DOI:** 10.1038/s41598-019-44273-5

**Published:** 2019-05-24

**Authors:** H. Dicko, O. Pagès, M. B. Shoker, F. Firszt, K. Strzałkowski, A. Maillard, A. Polian, Y. Battie, L. Broch, A. En Naciri, A. V. Postnikov, W. Paszkowicz, J.-P. Itié

**Affiliations:** 1 LCP-A2MC, Institut Jean Barriol, Université de Lorraine, Metz, F-57078 France; 2 Institute of Physics, N. Copernicus University, 87-100 Toruń, Poland; 3 LMOPS, Université de Lorraine – Supélec, 2 rue Edouard Belin, F-57070 Metz, France; 4 Institut de Minéralogie, de Physique des Matériaux et de Cosmochimie, Sorbonne Université —UMR CNRS 7590, F-75005 Paris, France; 5 Institute of Physics, Polish Academy of Sciences, Aleja Lotników 32/46, PL-02668 Warsaw, Poland; 6 Synchrotron SOLEIL, L’Orme des Merisiers Saint-Aubin, BP 48, F-91192 Gif-sur-Yvette Cedex, France

**Keywords:** Polaritons, Semiconductors

## Abstract

Volume-phonon-polaritons (*VPP*’s) propagating at a light-in-vacuum-like speed are identified in the wurtzite-type Zn_0.74_Mg_0.26_Se mixed crystal by near-forward Raman scattering. Their detection is selective to both the laser energy and the laser polarization, depending on whether the ordinary (*n*_0_) or extraordinary (*n*_*e*_) refractive index is addressed. Yet, no significant linear birefringence (*n*_0_ ${\boldsymbol{\simeq }}$ *n*_e_) is observed by ellipsometry. The current access to ultrafast *VPP*’s is attributed to the quasi-resonant Raman probing of an anomalous dispersion of *n*_0_ due to impurity levels created deep in the optical band gap by oriented structural defects. The resonance conditions are evidenced by a dramatic enhancement of the Raman signals due to the polar modes. Hence, this work reveals a capacity for the lattice defects’ engineering to “accelerate” the *VPP*’s of a mixed crystal up to light-in-vacuum-like speeds. This is attractive for ultrafast signal processing in the terahertz range. On the fundamental side we provide an insight into the *VPP*’s created by alloying ultimately close to the center of the Brillouin zone.

## Introduction

Replacement of electrons by photons as the carrier of information, leading to the development of photonics besides electronics, is highly promising in view to ultimately accelerate the signal processing in matter. On this way one resorts to elementary crystal excitations able to couple with light. Plasmon-polaritons spanning large to small plasma pulsations offer an option for data processing in the visible to terahertz spectral ranges^1–3^. In the latter range, the phonon-polaritons (*PP*) resulting from the coupling between optical lattice vibrations (phonons) and a photon-like (*i*.*e*., transverse) electric field $(\overrightarrow{E})$ in polar dielectrics are the other option^4^.

The existence of *PP*’s in common high-purity and high-structural-quality materials for electronics, *e*.*g*., the III-V and II-VI binary semiconductor compounds with zincblende (cubic) structure, has been known for decades^5–7^. The pioneering study of the *PP* coupling in GaP (III-V) compound done by near-forward (‘transmission’-like) Raman scattering goes back to the sixties^8,9^. The interest is revived at each novel generation of semiconductors; nowadays the focus has shifted onto the emerging class of N- based (III-V) compounds^10,11^. The physics behind the *PP*’s in pure compounds is well understood. Basically the *PP*’s can be classified into surface (*SPP*, ${\varepsilon }_{r}(\omega ) < 0$) or volume (*VPP*, ${\varepsilon }_{r}(\omega ) > 0$) ones depending on the sign of the relative dielectric function ${\varepsilon }_{r}(\omega )$, which changes on crossing the *Reststrahlen* $({\omega }_{TO}-{\omega }_{LO})$ band spanning the transverse (*TO*) and longitudinal (*LO*) pulsations of an optical phonon^12^.

In view of applications, the *SPP*’s are preferred over the *VPP*’s, because the propagation of the electromagnetic energy at the surface is less prone to dissipative effects due to defects and impurities^13,14^. Moreover, the confinement of light at the surface offers a possibility to achieve light localization into volumes down to a few nanometers, i.e. at a length scale much below the diffraction limit^1,15^. This is promising in view of optical signal processing in miniaturized devices at a level of integration comparable to that achieved in modern electronics. However, the *SPP* is bound to a fixed pulsation inside the *Reststrahlen* band, given by ${\varepsilon }_{r}({\omega }_{SPP})=-\,\,1$, that leads to high spectral selectivity. In contrast, the *VPP* is naturally dispersive. However, a major problem is that the *VPP* is hardly supported by a pure crystal since phonon decoupling occurs as soon as the *VPP* enters the highly-dispersive/high-velocity regime. The main features of the *SPP*’s and *VPP*’s related to a zincblende-type compound, corresponding to an unique optical phonon, are summarized in the Supplementary Section S1 for reference purpose, using ZnSe (II-VI) as a case study.

Elegant ‘extrinsic’ means to circumvent the absence of dispersion of the *SPP* are nanoarchitectural design or hybridization with a plasmon at metal/semiconductor-like interfaces^16,17^. An alternative ‘intrinsic’ option likely to reconcile the advantages of the *SPP* (signal processing at light-like speeds) and *VPP* (dispersive character) might be to (re)consider the *PP* coupling in volume, albeit in mixed crystals (multi-phonon systems) rather than in pristine (sole-phonon) ones.

**Figure 1:**
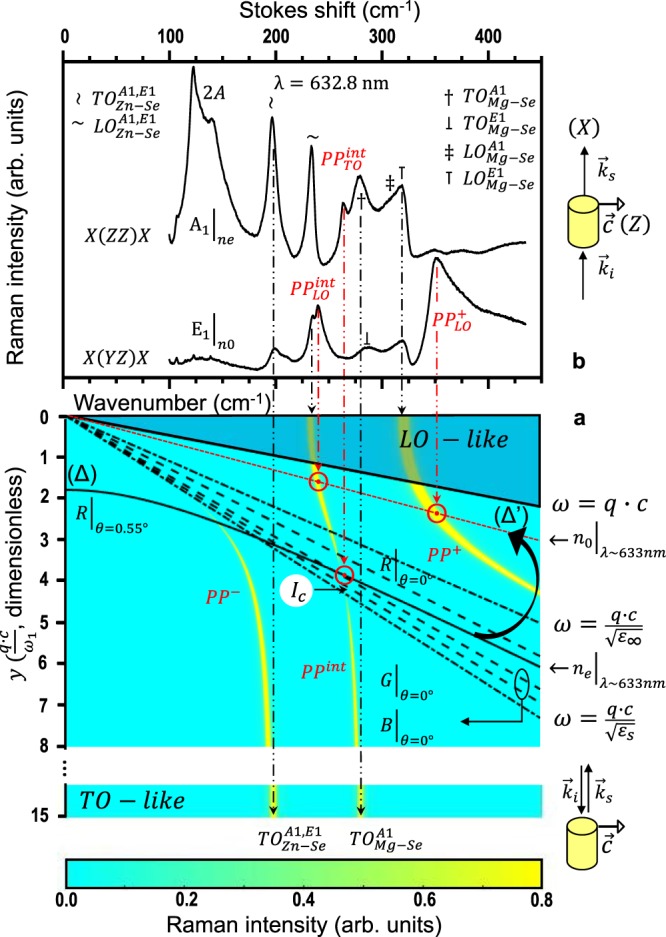
Near-forward-scattering Raman study of the Zn_0.74_Mg_0.26_Se *VPP*’s. (**a**) The reference *A*_1_–like dispersion of *VPP*’s (clear curves) including the *y*-dependence of the Raman intensity (thickness of the curves). (**b**) Polarized phonon-polariton Raman spectra in the near-forward (*θ* ~ 0°) *X*(*ZZ*)*X* and *X*(*YZ*)*X* scattering geometries. In panel (a), asymptotic regimes are defined by the dispersions of light well-beneath and well-beyond the phonon resonances (dashed-dotted curves, dictated by ${\varepsilon }_{s}$ and ${\varepsilon }_{\infty }$, respectively) and by the *LO*’s and *TO*’s close to ${\rm{\Gamma }}$ and away from ${\rm{\Gamma }}$, respectively. *I*_*c*_ refers to the photon-like extinction of the *PP*^*int*^ mode. The dispersion within the darkened area close to ${\rm{\Gamma }}$ has no physical meaning. The scan lines achievable experimentally by using the effective refractive index (*n*_*eff*_) in the perfect forward scattering geometry (*θ* = 0°) with the red (R, 632.8 nm), green (G, 514.5 nm) and blue (B, 488.0 nm) laser lines are indicated (dashed curves), for reference purpose. The scan line corresponding to the experimental *A*_1_–like *X*(*ZZ*)*X* Raman spectrum displayed in panel (b) is shown (*θ* = 0.55°). The curved arrow indicates an apparent upward-tilt of the corresponding scan line when shifting to the *E*_1_–like *X*(*YZ*)*X* geometry.

Such option, so far unexplored, may seem doomed to fail as the chemical and positional disorders inherent to a mixed crystal are expected to aggravate the dissipative effects that are nearly prohibitive already for pure crystals. In fact, we are not aware of any mixed crystal being considered so far for applications in photonics. Half a century separates the original near-forward Raman study of the *PP* coupling in GaP^9^ (still arising interest^18^) from the pioneering experimental works on the mixed crystals, engaged over the past decade. Nevertheless, several attractive features already emerge, summarized below, that might stimulate an interest in photonics for using the *VPP* created by alloying. The *ω*–dependent *VPP* Raman cross section shown in Fig. 1a for the particular mixed crystal studied in this work can be considered as generic, and thus used as a visual support to fix ideas in the following.

Calculations of the *VPP* dispersion via the Maxwell’s equations^8,19,20^ have shown that AB_1-x_C_x_ alloying generates an intermediary *VPP* with (A-B, A-C)-mixed character, i.e. *PP*^*int*^, between the *PP*^−^ and *PP*^+^ parent-like ones mostly related to the soft/heavy (say A-B) and stiff/light (A-C) bonds, respectively. *PP*^*int*^ differs in nature from *PP*^−^ and *PP*^+^ in that it exhibits a characteristic dispersion with a *S*-like shape between the *Reststrahlen* bands of the two bonds governed by two horizontal phonon asymptotes, and not by one phonon asymptote and one photon asymptote as is the case for the *PP*^−^ and *PP*^+^ dispersions. These two phonon asymptotes are positioned, away from the center ${\rm{\Gamma }}$ of the Brillouin zone $(q=0)$, at the pulsation of the upper *TO* mode (*TO*_*A*–*C*_), and, near ${\rm{\Gamma }}$, at the pulsation of the lower *LO* mode (*LO*_*A*–*B*_). The *S*-like dispersion can thus be adjusted to suit the user’s needs depending on the choice of parent compounds and on the crystal composition.

On the experimental side, pioneering far-infrared reflectivity studies of the *PP* coupling in thin films of wurtzite-type GaN-based mixed crystals were concerned with the *SPP* only^20,21^. Regarding the *VPP*, we are only aware of our own near-forward Raman studies on two zincblende-type ZnSe-based mixed crystals, namely Zn_1-x_Be_x_Se and ZnSe_1-x_S_x_ (refs^22–26^). The *Reststrahlen* bands of the parent compounds are well separated in these systems, so that the *PP*^*int*^ mode exhibits a large *S*-like dispersion, covering as much as ~200 cm^−1^ (~6 THz) in the case of Zn_1-x_Be_x_Se (see, e.g., ref.^26^), and shows up as a distinct feature in the Raman spectra. As such, the *PP*^*int*^ mode could be studied experimentally in detail.

The *S*-dispersion was to a certain extent probed by slightly varying the scattering angle *θ* between the incident $({\overrightarrow{k}}_{i})$ and scattered $({\overrightarrow{k}}_{s})$ light wavevectors inside the crystal near the perfect forward scattering geometry (*θ* = 0°), and also by changing the laser pulsation $({\omega }_{i})$. This latter option implies the dispersion of the refractive index $n(\omega )$ around ${\omega }_{i}$ that is decisive to how far (with respect to the *ω*_*min*_ value achieved at *θ* = 0°) a given laser line would ‘penetrate’ downward the *S*-shaped *PP*^*int*^ dispersion towards ${\rm{\Gamma }}$ (ref.^8^). The probing of the *S*-dispersion has revealed several attractive features of the *PP*^*int*^ mode:

(i) The *PP*^*int*^ Raman signal disappears at a critical point *I*_*c*_$({q}_{c},{\omega }_{c})$ near the *S*-inflexion, where the *PP*^*int*^ mode becomes photon-like. It is revived on both sides of *I*_*c*_, as the *PP*^*int*^ mode recovers a dominant phonon character. This conforms to intuition that only matter-like (phonon-like) excitations scatter light efficiently^25–27^.
(ii) On both sides of *I*_*c*_, the *PP*^*int*^ mode obeys the same Raman selection rules as its native purely-mechanical *TO* phonons away from ${\rm{\Gamma }}$ (ref.^26^). This means that the *PP*^*int*^ mode preserves its transverse character throughout its entire *S*-like dispersion, even in the *LO*-like asymptotic regime near ${\rm{\Gamma }}$. As such, it keeps an ability to couple strongly with light at any stage.
(iii) The agreement between the experimental and theoretical *PP*^*int*^ selection rules is excellent ante $(\omega  > {\omega }_{c})$ and post $(\omega  < {\omega }_{c})$ photon-like extinction^26^. Apparently the long wavelength in the *PP* regime $(q\sim 0)$ operates a natural average on the alloy disorder, so that the *PP*^*int*^ mode does ‘see’ neither the chemical disorder nor the local lattice distortions inherent to a mixed crystal, and propagates like in a perfect medium.
(iv) While the *PP*^*int*^ mode ante-extinction exhibits a large Raman linewidth comparable to that of its native *TO* phonon away from ${\rm{\Gamma }}$, it becomes sharp post-extinction while approaching ${\rm{\Gamma }}$ (refs^25,26^) – an explanation is given in the Supplementary Section S4, with concomitant increase on the *PP*^*int*^ lifetime^28^. This opposes to the experimental trend in a pure crystal (a direct insight in the case of ZnSe is given in the Supplementary Fig. S2)^8^.

Summarizing, the naturally dispersive *PP*^*int*^ mode created by alloying is interesting in many respects: (i) it remains supported by the crystal (phonon-like) throughout most of its *S*-like dispersion, (ii) it is likely to couple with light throughout its whole dispersion, (iii) it ‘feels’ like propagating in a pure crystal, and, beyond all expectation, (iv) it gains lifetime while acquiring high velocities ($q\to 0$). Of upmost interest in view of applications is the latter ‘high-velocity/long-lifetime’ regime of the *PP*^*int*^ dispersion situated beneath *I*_*c*_. So far, only its early stage near *I*_*c*_ could be probed experimentally, leaving the lower part of the *S*-like dispersion totally unexplored across all studied mixed crystals^22–26^. An access near ${\rm{\Gamma }}$ is needed to complete the *PP*^*int*^ picture in view of applications, but also for the sake of fundamental understanding. As already mentioned, the limiting factor is the finite dispersion of the refractive index *n*$(\omega )$ around ${\omega }_{i}$ that so far hindered to approach sufficiently small *q* values.

In this work, the lacking insight into the *PP*^*int*^ mode near ${\rm{\Gamma }}$ is searched for by trying near-forward Raman scattering on a wurtzite-type ZnSe-based mixed crystal, using Zn_0.74_Mg_0.26_Se as a case study. The ZnSe-based crystals are transparent in the visible (the optical band gap of ZnSe is 2.7 eV at room temperature^29^) and thus well-suited for a Raman study in ‘transmission’. Moreover, owing to a large difference in the Zn (~65) and Mg (~24) atomic masses, the *Reststrahlen* bands of the wurtzite-type ZnSe^30^ and MgSe^31^ compounds are well separated, i.e. by ~20 cm^−1^, which is needed for the *PP*^*int*^ mode to exhibit a pronounced *S*-like dispersion. This separation is preserved with alloying^32–34^. Last but not least, Zn_0.74_Mg_0.26_Se is interesting for its wurtzite structure – in part because the *PP*^*int*^ mode remained unexplored in anisotropic crystals so far, but moreover in a hope that the vibrational and optical anisotropies behind the structural anisotropy would help to diversify the access to *PP*^*int*^. Indeed, for each phonon that is likely to support the *VPP* coupling, in reference to the *A*_1_ and *E*_1_ polar vibrations along and perpendicular to the $\overrightarrow{c}$-crystal axis, respectively^10,11^, the *PP*^*int*^ insight can be optimized by playing with the dispersion of either the ordinary (*n*_0_) or extraordinary (*n*_*e*_) refractive index. Altogether the access to the *PP*^*int*^ mode by near-forward Raman scattering is thus potentially quadruple (2 phonons × 2 refractive indices) with a wurtzite-type system such as Zn_0.74_Mg_0.26_Se, and not only unique (1 phonon × 1 refractive index) as with the zincblende-type ZnSe-based mixed crystals studied so far. The opportunities to capture novel information near ${\rm{\Gamma }}$ are correspondingly enriched.

## Results and Discussion

No significant linear birefringence could be detected by ellipsometry in the visible where operates the Raman scattering. An effective refractive index ${n}_{eff}(\omega )$ is thus considered from now on for Zn_0.74_Mg_0.26_Se. Besides, in the Zn_0.74_Mg_0.26_Se near-forward Raman spectra taken with the red laser line (632.8 nm, Fig. 1b), the native Zn-Se (~200 cm^−1^) and Mg-Se (~280 cm^−1^) purely-mechanical *TO*’s of the *VPP*’s, and also the related *LO*’s, both theoretically forbidden but activated due to multi-reflection of the laser beam between faces of the crystal^9^, appear to be quasi degenerate in the *A*_1_ and *E*_1_ symmetries (within ~5 cm^−1^). Altogether, the quasi negligible linear birefringence $({n}_{0}\simeq {n}_{e})$ and the apparent *A*_1_ − *E*_1_ degeneracy ruin all hopes of achieving a diversified *PP*^*int*^ insight by playing with the optical and vibrational anisotropies behind the wurtzite structure of Zn_0.74_Mg_0.26_Se, independently evidenced by X-ray diffraction. Details concerning the ellipsometry and X-ray diffraction measurements are given in the Supplementary Section S2.

Based on the observed pulsations of the Zn-Se and Mg-Se purely-mechanical *TO*’s, an overview of the *VPP* dispersion is achieved by solving numerically

1 $${Im}\{\frac{-1}{{\varepsilon }_{r}(\omega )-{q}^{2}\cdot {c}^{2}\cdot {\omega }^{-2}}\}=0,$$

that captures the resonances behind the dispersion of a *TO* mode, using a classical two-oscillator $[1\times (Zn-Se),1\times (Mg-Se)]$ form for ${\varepsilon }_{r}(\omega )$. A more refined expression in which the resonance-term is weighted by a pre-factor involving the Faust-Henry coefficients of ZnSe and MgSe, derived in ref.^22^, provides the actual *VPP* Raman cross section in its *ω*–dependence, shown in Fig. 1a. Note the punctual extinction (at *I*_*c*_) of the *PP*^*int*^ Raman signal in the photon-like regime. Detail is given in the Supplementary Section S4.

The near-forward Raman spectrum taken in the *X*(*ZZ*)*X* pure–*A*_1_ geometry (Fig. 1b), using the Porto’s notation^35^, reveals a sharp *PP*^*int*^ mode in the shallow reinforcement regime (*ω* ~ 260 cm^−1^ < *ω*_*c*_ ~ 265 cm^−1^). The corresponding Raman ‘scan’ line (*θ* ~ 0.55°) in Fig. 1a, as derived from the wavevector conservation law that governs the Raman scattering $({\overrightarrow{k}}_{i}-{\overrightarrow{k}}_{s}=\overrightarrow{q})$ using the effective refractive index measured by ellipsometry (see the Supplementary Section S1) to express the magnitudes of ${\overrightarrow{k}}_{i}$ and ${\overrightarrow{k}}_{s}$, crosses the *S*-like *PP*^*int*^ dispersion exactly between ${\omega }_{c}$ (*θ* ~ 0.80°) and *ω*_*min*_ (~258 cm^−1^, *θ* = 0°). As the *PP*^*int*^ mode emerges near its native $T{O}_{Mg\mbox{--}Se}^{A1}$ mode, it is referred to as $P{P}_{TO}^{int}$. As soon as *θ* increases, the $P{P}_{TO}^{int}$ mode vanishes. This is consistent with its entry into the photon-like extinction regime $(\omega \sim {\omega }_{c})\,$on its way back to the collapse regime $(\omega  > {\omega }_{c})$.

The $X(ZZ)X\to X(YZ)X$ shift of geometry of the $({n}_{e},{A}_{1})\to ({n}_{0},{E}_{1})$ type, obtained by rotating the laser polarization, truly shakes the *PP* signal, with no obvious explanation since the linear birefringence is quasi negligible and the native (*A*_1_, *E*_1_) phonons behind the *PP*’s are quasi degenerate. Beyond all expectations, the *PP* dispersion is now probed in its deep reinforcement regime close to ${\rm{\Gamma }}$, where both the *PP*^*int*^ and *PP*^+^ modes assimilate with their asymptotic *LO*_*Zn*–*Se*_ and *LO*_*Mg*–*Se*_ limits $(\omega \ll {\omega }_{c})$, respectively, thus labelled as $P{P}_{LO}^{int}$ and $P{P}_{LO}^{+}$. It is as if by rotating the incident polarization the Raman ‘scan’ line (Δ′) was tilted upward its nominal position (Δ) dictated by *n*_*eff*_$(\omega )$ close by the dispersion of light in vacuum (Fig. 1a, refer to the curved arrow).

Neither the $P{P}_{TO}^{{int}}$ mode nor the $(P{P}_{LO}^{{int}},P{P}_{LO}^{+})$ ones survive when adopting the *X*(*ZY*)*X* and *X*(*YY*)*X* geometries, for different reasons. The former geometry probes the *E*_1_-like counterpart of $P{P}_{TO}^{{int}}$ in the final stage of its collapse regime where the mode is overdamped. In the *X*(*YY*)*X* geometry the *E*_1_ modes are forbidden while the *A*_1_ ones are marginally activated and anyway screened by spurious features, with concomitant impact on the related *PP*’s. Detail is given in the Supplementary Section S4.

**Figure 2:**
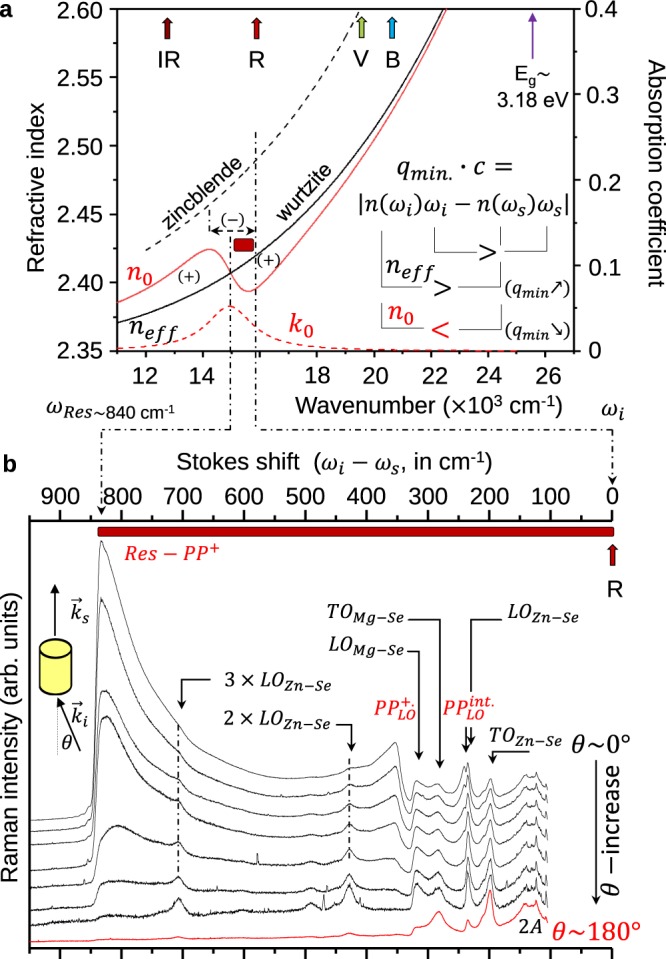
Resonance-induced near-forward Raman scattering – impact on the dispersion of the refractive index. (**a**) Measured dispersion of the effective refractive index of the wurtzite-type Zn_0.74_Mg_0.26_Se mixed crystal (solid line) in the visible (400–800 nm). The dispersion for the corresponding zincblende-type system (taken from ref.^37^) is added (dotted curve) for comparison. The resonance at ${\omega }_{{Res}}$ creates a local absorption (*k*_0_) in the red spectral range eventually leading to a local inversion (−) of the nominally positive (+) dispersion of *n*_0_ near ${\omega }_{i}$. This inversion offers a chance to achieve extremely small *q*_*min*_ values, as schematically explained in the inset, corresponding to an upward-tilt of the nominal scan line (curved arrow in Fig. 1a). The used near-infrared (NIR, 785.0 nm), red (R, 632.8 nm), green (G, 514.5 nm) and blue (B, 488.0 nm) laser lines are indicated. (**b**) Expanded *A*_1_–like near-forward Raman spectra (*θ* ~ 0°) taken with the red laser line at increasing *θ* values (from top to bottom) until disappearance of the *VPP*’s. The enhancement of the polar (*VPP*, *LO*)–like Raman signals is due to the resonance. The Raman spectrum taken in the backscattering geometry (*θ* ~ 180°) is added for reference purpose.

The ${\rm{\Delta }}\to {\rm{\Delta }}^{\prime}$ upward-tilt needed to mimic the apparent reduction of ${q}_{{\min }}={c}^{-1}\cdot |{n}_{eff}({\omega }_{i})\cdot {\omega }_{i}-{n}_{eff}({\omega }_{s})\cdot {\omega }_{s}|$ in the *X*(*YZ*)*X* geometry can be explained only if the difference in pulsations $|{\omega }_{i}-{\omega }_{s}|$ is countered by the corresponding difference in indices $|{n}_{eff}({\omega }_{i})-{n}_{eff}({\omega }_{s})|$, and not emphasized by the latter as expected in view of the positive dispersion of ${n}_{eff}(\omega )$ measured by ellipsometry (see Methods). The tilt suggests an anomalous dispersion of *n*_0_ near ${\omega }_{i}$, presumably due to impurity levels created by structural defects. These give rise to a local absorption reflected by a maximum in ${Im}\{{\varepsilon }_{r}(\omega )\}$ (as sketched out in Fig. 2a – dotted curve) going with a sigmoidal distortion of ${Re}\{{\varepsilon }_{r}(\omega )\}$, with concomitant impact on the dispersion of *n*_0_, being inverted locally (thick curve) – basic relations are given, e.g., in ref.^36^. In Fig. 2a, the dispersion of the refractive index for the zincblende-type Zn_0.74_Mg_0.26_Se single-crystalline epitaxial film^37^ is added for comparison.

In fact, the activation of the $P{P}_{LO}^{{int}}$ and $P{P}_{LO}^{+}$ modes in the *X*(*YZ*)*X* geometry is selective to ${\omega }_{i}$. It is strong with the red (632.8 nm) laser line and also, to a less extent, with the near-infrared (785.0 nm) one, but remains weak with the green (514.5 nm) and blue (488.0 nm) ones (examples are given in the Supplementary Fig. S10). This suggests a resonance mechanism involving impurity levels mostly situated deep in the optical band gap. The resonance is searched for by extending the Raman analysis away from ${\omega }_{i}$ using the red laser line (Fig. 2b). A typical two-feature set, combining a dramatic enhancement of the Raman signal preceding its sudden extinction at ~840 cm^−1^ beneath ${\omega }_{i}$ (considering the used Stokes scattering), reveals a resonance with an impurity level positioned right at the extinction (*i*.*e*., at ${\omega }_{{Res}}\sim {\omega }_{i}-\,840\,{{\rm{cm}}}^{-1}$). The enhanced signal is of the *PP* type since it vanishes along with the $P{P}_{LO}^{{int}}$ and $P{P}_{LO}^{+}$ modes when *θ* increases (Fig. 2b). It is assigned as a resonance-induced *PP*^+^ mode (*Res* − *PP*^+^) away from the *LO*-like limit of the *PP*^+^ dispersion. At large scattering angle (*θ* ≥ 2°) the *PP*’s disappear. The only left polar ($\overrightarrow{E}$–equipped) modes likely to be resonantly enhanced (via the $\overrightarrow{E}$–mediated Fröhlich mechanism^38^) are then the *LO* ones, notably the sharp and intense *LO*_*Zn*–*Se*_ peak, giving rise, in fact, to resonance-activated harmonics up to the third order.

Earlier photoluminescence measurements done with Zn_1-x_Mg_x_Se (0 < *x* < 0.6) monocrystals from the same source as the current one (*x* = 0.26) have revealed a strong red emission, providing a direct evidence for a continuum of impurity levels deep in the optical band gap^39^. Based on positron annihilation measurements, the red emission has been related to Zn vacancies (*V*_*Zn*_), possibly arranged as dimers^39^. As the activation of the $P{P}_{LO}^{{int}}$ and $P{P}_{LO}^{+}$ Raman features occurs only when ${\overrightarrow{e}}_{i}\parallel \overrightarrow{c}$ and in a given phonon (*E*_1_) symmetry, we conclude that the *V*_*Zn*_–related complexes have a preferential orientation with respect to the $\overrightarrow{c}$–axis of the crystal. The concentration of such complexes is small, though, since no singularity is detected in the red spectral range of the linear dichroism measured by transmission ellipsometry (see the Supplementary Fig. S4). Their massive impact on the *X*(*YZ*)*X* Raman spectra is all due to the resonance conditions.

Technically, a crude contour modeling of the bimodal ($P{P}_{LO}^{{int}}$, $P{P}_{LO}^{+}$) Raman signal can be achieved (see the Supplementary Fig. S9) by considering that the distorted dispersion of the refractive index in the studied ${\omega }_{{Res}}\le {\omega }_{s}\le {\omega }_{i}$ spectral domain (emphasized in Fig. 2a) is opposite (negative) and roughly twice as large in magnitude as that (positive) measured for ${n}_{eff}(\omega )$ by ellipsometry. To fix ideas, the amount of oscillator strength (taken as the only adjustable parameter – see details in the Supplementary Section S4) needed to achieve such inversion (shown in Fig. 2a) by introducing one unique damped Lorentz oscillator at *ω*_*Res*_ was estimated at ~1% of the oscillator strength carried by the optical phonon of ZnSe.

Summarizing, the Zn_0.74_Mg_0.26_Se mixed crystal with wurtzite structure is studied by near-forward Raman scattering. Despite the almost negligible linear birefringence $({n}_{0}\sim {n}_{e})$ observed by ellipsometry, an unexpectedly large variety of *VPP*’s is revealed, contrasting with the quasi-indiscernibility of the native *A*_1_ and *E*_1_ phonons. This is due to an artificial linear birefringence created by deep impurity levels associated with oriented crystal defects. The artificial linear birefringence grants access to the *PP*^*int*^ mode both in its shallow reinforcement regime just beneath the photon-like extinction *I*_*c*_ (${\overrightarrow{e}}_{i}\parallel \overrightarrow{c}$) and far from *I*_*c*_ deep in its reinforcement regime ultimately close to ${\rm{\Gamma }}$ (${\overrightarrow{e}}_{i}\perp \overrightarrow{c}$), where it acquires a light-in-vacuum-like speed. An added bonus in this case is the access to *PP*^+^. On the practical side, the easy-to-handle shift from the shallow $(P{P}_{TO}^{{int}})$ to deep $(P{P}_{LO}^{{int}})\,$reinforcement regimes of *PP*^*int*^ by merely rotating the laser polarization provides, in fact, a sharp $(P{P}_{TO}^{{int}}-P{P}_{LO}^{{int}})\,$optical switch, with potential applications in photonics.

## Methods

This Sec. introduces the experimental methods needed for interpretation and replication of the reported data in the main part of the manuscript. Additional experimental and theoretical aspects, covering X-ray diffraction, ellipsometry measurements done in transmission, conventional backward Raman scattering, *ab initio* calculations on the native phonons behind the phonon-polaritons, are given in the course of the discussion of the corresponding data in the Supplementary Sections S2 and S3.

### Sample growth and preparation

The used sample consists of a large high-quality (Zn,Mg)Se single crystal (cylinder, 3 mm in height and 8 mm in diameter) with ‘pure’ wurtzite structure, grown by the Bridgman method. The composition estimates from the *a* and *c* lattice constants measured by X-ray diffraction at the PSICHÉ beamline of synchrotron SOLEIL are 26.2 and 25.9 at.%Mg, respectively. Detail is given in the Supplementary Section S2. A fragment (~2.5 mm in length) of the crystal was oriented by conoscopy, using a cross-polarized microscope, so as to dispose of one pair of parallel (within 1°) faces (~0.5 mm^2^, giving rise to hyperbolic fringes by conoscopy) with in-plane $\overrightarrow{c}$–axis (within 5°), plus one face perpendicular to the $\overrightarrow{c}$–axis (~2 mm^2^, circular fringes). The three faces were polished to optical quality in view of polarized Raman measurements.

### Ellipsometry measurements

The dispersion of the refractive index of the main non-oriented crystal measured by conventional ellipsometry (using a HORIBA UVISEL phase modulated spectroscopic ellipsometer) at a near-Brewster incidence in the visible spectral range, where operates the Raman scattering, does not reveal any sign of linear birefringence (*LB*). The measured dispersion of the refractive index, hence an effective one, noted *n*_*eff*_, was found to obey the Cauchy’s formula ${n}_{eff}(\lambda )=X+Y\cdot {\lambda }^{-2}\cdot {10}^{-4}+Z\cdot {\lambda }^{-4}\cdot {10}^{-9}$ with $X=2.3524\mp 0.0021$, $Y=0.4336\mp 0.0084\,n{m}^{2}$ and $Z=8.9110\mp 0.1772\,n{m}^{4}$ (with *λ* in *nm*). More refined ellipsometry measurements done in transmission through the small $\overrightarrow{c}$–containing faces of the oriented fragment at normal incidence (see the Supplementary Fig. S4), treated within the approach of the Mueller’s matrix^36^, revealed that our Zn_0.74_Mg_0.26_Se crystal is, in fact, uniaxial positive $[{n}_{e}(\omega ) > {n}_{o}(\omega )]$. However, for our use at least, the ${n}_{e}(\omega )\sim {n}_{o}(\omega )$ equivalence basically applies. More precisely, for the considered Zn_0.74_Mg_0.26_Se crystal the linear birefringence is constant and presumably smaller than 0.020 (detail is given in the Supplementary Section S2). Whether considering that *n*_*e*_$(\omega )$ is step-increased/decreased from ${n}_{eff}(\omega )$ by ±0.02 throughout the visible we have checked that the ‘scan’ lines achieved by Raman scattering at near-normal incidence (*θ* = 0.55°) with the used (R) laser line polarized perpendicular to $\overrightarrow{c}$ (the relevant refractive index is *n*_0_, then) superimpose exactly, *i*.*e*., the variation remains within the thickness of the ‘scan’ line (Fig. 1a).

### Near-forward Raman measurements

Near-forward Stokes (${\omega }_{i} > {\omega }_{s}$) Raman spectra are taken by focusing the exciting laser beam at normal incidence onto the rear crystal face containing the $\overrightarrow{c}$– crystal axis. The scattered light originates from the focus plane of the incident lens, and is detected in the same direction as the incident laser beam, *i*.*e*., normally to the front crystal face. The limiting factor to achieve the perfect forward scattering geometry (*θ* = 0°) is the numerical aperture of the microscope objective used to collect the scattered light, corresponding to a moderate focal length (~3.00 cm, slightly depending on the used laser line) and a significant diameter (0.40 cm). In contrast the incident laser beam has a small diameter (0.20 cm) and moreover the focal length of the incident lens is large (~15.00 cm). With this, the scattered light detected outside the sample fits into a pencil-like 3.80° solid cone, to compare with a narrower 0.35° solid cone for the exciting laser beam.

Polarized near-forward Raman spectra are taken by placing half-wave plates on each side of the crystal with their neutral axis disposed either parallel or rotated by 45° to each other and with respect to the $\overrightarrow{c}$-crystal axis. The scattered light is analyzed parallel to the entrance slit of the spectrometer corresponding to maximum spectrometer throughput. The as-obtained scattering geometries with parallel and crossed polarizations of the incident laser (${\overrightarrow{e}}_{i}$) and scattered light (${\overrightarrow{e}}_{s}$) write *X*(*ZZ*)*X*, *X*(*ZY*)*X*, *X*(*YZ*)*X* and *X*(*YY*)*X* using Porto’s notation ${\overrightarrow{k}}_{i}({\overrightarrow{e}}_{i},{\overrightarrow{e}}_{s}){\overrightarrow{k}}_{s}$ (ref.^35^). The *X* and *Z* axis of the laboratory coordinate system coincide with the directions of the incident laser (${\overrightarrow{k}}_{i}$) and of the scattered light (${\overrightarrow{k}}_{s}$) and with the $\overrightarrow{c}$ – crystal axis, respectively. Both *X*(*ZZ*)*X* and *X*(*YY*)*X* consist of ‘pure’ *A*_1_-like geometries for the *TO* modes (the *VPP*’s as well as their native *TO*’s, depending on whether the analysis is placed near ${\rm{\Gamma }}$ using near-forward Raman scattering or away from it as in a backscattering Raman experiment, respectively). Nevertheless the *A*_1_–TO Raman signal shows up clearly only in the first geometry. It is negligible in the second one (hence not shown). In contrast the *X*(*ZY*)*X* and *X*(*YZ*)*X* geometries are of the pure *E*_1_ type. The only difference is that in the former geometry the incident laser beam propagates as an extraordinary wave (${\overrightarrow{e}}_{i}\parallel \overrightarrow{c}$) inside the crystal whereas as an ordinary one (${\overrightarrow{e}}_{i}\perp \overrightarrow{c}$) in the latter geometry, the outgoing scattered light being ordinary (${\overrightarrow{e}}_{s}\perp \overrightarrow{c}$) in both cases. When needed (Fig. 2b), the departure from the nominally perfect forward scattering geometry (*θ* = 0°) was operated by finely adjusting the incidence of the laser beam in the (*x*, *y*) plane, while detecting the scattered light along the fixed *X* direction.

The theoretical dependence of the crossed- and parallel-polarized *A*_1_ and *E*_1_ *TO* Raman intensities on the azimuth angle (*α*) between the incident polarization and the $\overrightarrow{c}$–crystal axis for a complete *α*-revolution at the sample surface, calculated by using the relevant wurtzite-type Raman tensors, is reported in the Supplementary Fig. S5, for reference purpose.

## Supplementary information

Defect-induced ultimately fast volume phonon-polaritons in the wurtzite Zn<sub>0.74</sub>Mg<sub>0.26</sub>Se mixed crystal

## Data Availability

All data regarding the work presented here including the Matlab routine for contour modeling of the Raman cross section of the *VPP* modes in their $(\theta ,y,\omega )$–dependence is available upon request to the corresponding author.
