# Supplementary material for: Defect-induced ultimately fast volume phonon-polaritons in the wurtzite Zn0.74Mg0.26Se mixed crystal
Source: Sci Rep. 2019 May 24;9:7817. doi: 10.1038/s41598-019-44273-5 (PMC6534573; doi:10.1038/s41598-019-44273-5)
Supplement: Supplementary file 1 — Defect-induced ultimately fast volume phonon-polaritons in the wurtzite Zn0.74Mg0.26Se mixed crystal [file 41598_2019_44273_MOESM1_ESM.pdf]

Supplementary Information

Defect-induced ultimately fast volume phonon-polaritons in the wurtzite $Zn_{0.74}Mg_{0.26}Se$ mixed crystal

H., Dicko, O., Pagès, M. B., Shoker, F., Firszt, K., Strzałkowski, A., Maillard, A., Polian, Y., Battie, L., Broch, A., En Naciri, A. V., Postnikov, W., Paszkowicz & J.-P. Itié

This section, concerned with side aspects of the VPP modes detected by near-forward Raman scattering with the wurtzite-type (hexagonal) $Zn_{0.74}Mg_{0.26}Se$ mixed crystal, is organized as follows. Sec. 1 gives basics on the dispersions of the VPP (Sec. 1.1a) and the SPP (Sec. 1.1b) modes in a semiconductor compound (Sec. 1.1) and their detection by Raman scattering (Sec. 1.2). The relative merits of the backward and forward scattering geometries are discussed (Sec. 1.2a), supported by experimental data (Sec. 1.2b) obtained with the pure zincblende-type (cubic) ZnSe crystal, constituting a natural reference for the current study of $Zn_{0.74}Mg_{0.26}Se$. Sec. 2 reports on the sample growth (Sec. 2.1), and on its structural, optical and basic vibrational (not VPP-like) properties, as measured by X-ray diffraction (Sec. 2.2), by transmission spectroscopic ellipsometry (Sec. 2.3) and by Raman scattering operated in the conventional backscattering geometry (Sec. 2.4) giving access to the native purely mechanical TO's behind the VPP's. The discussion of the latter modes is supported in Sec. 3 by an exhaustive *ab initio* insight (Sec. 3.1) into the vibration properties of prototypal impurity motifs immersed in large (64-atom) supercells (Sec. 3.2) offering a possibility to derive a wurtzite-type version of the percolation scheme for $Zn_{1-x}Mg_xSe$ (Sec. 3.3). Sec. 4 lays the foundations for the discussion of the PP^{int} mode due to alloying in the main part of the manuscript. Reference theoretical insights into the VPP dispersion of $Zn_{0.74}Mg_{0.26}Se$ and into the related (y,θ)-dependencies of the Raman intensities obtained are obtained within the linear dielectric response theory (Sec. 4.1). Near-forward Raman studies of the ZnSe-like PP^{-} (Sec. 4.2) and MgSe-like PP^{+} (Sec. 4.3) satellites of the main PP^{int} mode depending on the used laser excitation (ω_i) and/or on the scattering angle (θ) complete the VPP picture of $Zn_{0.74}Mg_{0.26}Se$.

1. Near-forward Raman insight into the VPP and SPP modes of the reference ZnSe crystal with zincblende structure

Prior to considering the detection of the VVP and SPP modes of the reference ZnSe compound with zincblende structure by near-forward Raman scattering (Sec. 1.2), we introduce briefly the main features of such modes (Sec. 1.1).

1.1. The PP modes of ZnSe. Due to the quasi vertical dispersion of light ($\omega = c \cdot q$ in vacuum, scaled down by the refractive index in ZnSe) the Raman scattering operates near the center Γ ($q \sim 0$, $\lambda \to \infty$) of the Brillouin zone, where only the optical lattice vibrations propagate. The relevant oscillator near Γ corresponds to the relative displacement $\vec{u}$ of the two intercalated cation (Zn) and anion (Se) sublattices taken as quasi rigid ones ($\lambda \to \infty$). Owing to their polar character, such "cation – vs – anion" vibrations are expected to carry a long range (macroscopic, $\lambda \to \infty$) electric field $\vec{E}$, governed by the Maxwell's equations. Such $\vec{X} = (\vec{u}, \vec{E})$ optical lattice vibrations may propagate as a volume mode or as a surface one. The corresponding dependencies on time t and position $\vec{r}$ of the effective bond stretching take the form of a plane wave $\vec{X} = \vec{X}_0 \cdot e^{j\cdot\{\omega\cdot t - \vec{q}\cdot\vec{r}\}}$ with wavevector $\vec{q}$ defined inside the crystal or of an evanescent wave $\vec{X} = \vec{X}_0 \cdot e^{j\cdot\{\omega\cdot t - \vec{q}_{\parallel}\cdot\vec{r}\}} \cdot e^{-\alpha|z|}$ where $\alpha \geq 0$, $|z|$ represents the distance to the surface, and $\vec{q}_{\parallel}$ is taken in-plane at the surface, respectively.

1.1-a. The VPP mode. By applying the Maxwell's equations to the plane wave (in reference to the volume mode) in an isotropic dielectrics (such as ZnSe), $\vec{E}$ takes the generic form (see detail, *e.g.*, in Ref. 1),

$$\vec{E} = \left[\frac{\omega^2}{c^2} \times \vec{p} - \vec{q} \times (\vec{q} \cdot \vec{p})\right] \times \left[\varepsilon_0 \times \left(q^2 - \frac{\omega^2}{c^2}\right)\right]^{-1}, \quad (1)$$

where $\vec{p}$ is the polarization (the electric dipolar moment per unit crystal volume, remaining parallel to $\vec{u}$ in a cubic crystal like ZnSe), ε_0 is the permittivity of vacuum and c is the speed of light in vacuum. The macroscopic electric field carried by the LO mode ($\vec{u} \parallel \vec{q} \parallel \vec{p}$), *i.e.*, $\vec{E}_L = -\frac{\vec{p}}{\varepsilon_0}$, is non dispersive, so that the LO pulsation is not q-dependent, noted ω_{LO}. In contrast the macroscopic electric field that equips the TO mode ($\vec{u} \perp \vec{q} \parallel \vec{p}$) is q-dependent, writing as

$$\vec{E}_T = \frac{\omega^2}{c^2} \times \left[\varepsilon_0 \times \left(q^2 - \frac{\omega^2}{c^2}\right)\right]^{-1} \times \vec{p}, \quad (2)$$

with concomitant impact on the TO pulsation, being also q-dependent, noted $\omega_T(q)$. Strictly at Γ, $\vec{E}_T(q=0) = \vec{E}_L$, meaning that the TO and LO modes are degenerated. Indeed at this limit the antiphase displacement of the perfectly rigid ($\lambda \to \infty$) cationic and anionic sublattices, characteristic of an optical mode at Γ, can indifferently be described in terms of a vibration along the wavevector $\vec{q}$ (LO) or perpendicular to it (TO) since q is null in fact. Away from Γ, $\vec{E}_T(q)$ collapses to zero. An intuitive explanation is that by departing from the center Γ of the Brillouin zone ($q \gg \frac{\omega}{c}$), the (ω, q) values for the TO mode fall so far away from the quasi vertical ($q \sim 0$) dispersion $\omega(q)$ of a photon – namely a pure transverse electric field – that they are not compatible with the propagation of a transverse electric field. The corresponding TO mode is thus deprived of electric field, reducing to a purely mechanical vibration. We refer to the pulsation of the corresponding purely-mechanical TO vibration, abbreviated TO hereafter, as ω_{TO}. By reducing q and approaching the quasi vertical dispersion of a photon, the transverse electric field of a TO mode is restored. The resulting TO excitation with mixed electrical–mechanical character is called a phonon-polariton (PP).

At this stage, two asymptotic limits are identified for the pulsation $\omega_T(q)$ of the TO mode, namely $lim_{q\to 0}\, \omega_T(q) = \omega_{LO}$ and $lim_{q\to\infty}\, \omega_T(q) = 0$. Below we are interested in the $\omega_T(q)$ dependence in the intermediary (ω, q) −domain.

By introducing the relative dielectric function $\varepsilon_r(\omega)$ of the crystal, according to $\varepsilon_0\varepsilon_r(\omega)\vec{E} = \varepsilon_0\vec{E} + \vec{p}$, the obtained expressions for $\vec{E}_L$ and $\vec{E}_T(q)$ above are recovered by considering that $\varepsilon_r(\omega) = 0$ for the LO mode and $\varepsilon_r(\omega) = \frac{q^2 \times \omega^2}{c^2}$ for the TO mode. As is well known (see Ref. 5 – main text, p. 118), for a mono-oscillator (in reference to the $TO - LO$ optical phonon) crystal such as ZnSe, $\varepsilon_r(\omega)$ takes the following form in the THz spectral range

$$\varepsilon_r(\omega) = \varepsilon_\infty + (\varepsilon_s - \varepsilon_\infty) \times \left(1 - \frac{\omega^2}{\omega_{TO}^2}\right)^{-1}, \quad (3)$$

where ε_s and ε_∞ define the relative dielectric constants at pulsations far-below ($\omega \ll \omega_{TO}$) and far-beyond ($\omega \gg \omega_{TO}$) the phonon resonance, respectively.

By applying $\varepsilon_r(\omega) = 0$ for the LO mode, the Lyddane-Sachs-Teller (LST) equation emerges, *i.e.*, $\frac{\varepsilon_s - \varepsilon_\infty}{\varepsilon_\infty} = \frac{\omega_{LO}^2 - \omega_{TO}^2}{\omega_{TO}^2}$, that basically relates the (relative) jump in dielectric constant $(\varepsilon_s - \varepsilon_\infty)$ occasioned by crossing the phonon resonance, commonly referred to as the oscillator strength of the phonon, to the (relative) magnitude of the *Reststhrahlen* $(\omega_{TO} - \omega_{LO})$ band representing that phonon resonance. As $\varepsilon_r(\omega)$ is generally a decreasing function of ω (reflecting a basic trend that the crystal polarization due to an externally-applied oscillating electric field enlarges when ω reduces), it follows from the LST relation that the LO mode is bound to a fixed pulsation situated above the $PM - TO$ one, given by $\omega_{LO}^2 = \omega_{TO}^2 \times \frac{\varepsilon_s}{\varepsilon_\infty}$. This conforms to intuition since the (non dispersive) macroscopic electric field carried by the LO mode generates a Coulombian restoring force on top of the elastic one characteristic of the purely-mechanical TO mode. The apparent restoring force constant is correspondingly enlarged for the LO mode, with concomitant impact on its pulsation, being higher than that of its TO counterpart.

The dispersion of the TO mode (including the PP and its native purely-mechanical TO as well), *i.e.*, $\varepsilon_r(\omega) = \frac{q^2\times\omega^2}{c^2}$, develops into a second-degree equation whose solutions are the following TO pulsations squared

$$\omega_T^2(q) = -\frac{1}{2}\left\{-\left(\omega_{LO}^2 - \frac{q^2\times c^2}{\varepsilon_\infty}\right) \mp \left[\left(\omega_{LO}^2 + \frac{q^2\times c^2}{\varepsilon_\infty}\right)^2 - 4\frac{q^2\times c^2}{\varepsilon_\infty}\omega_{TO}^2\right]^{\frac{1}{2}}\right\}. \quad (4)$$

These contain all information as how the TO pulsation changes while the transverse electric field $\vec{E}_T(q)$ develops at the approach of Γ. The asymptotic solutions for the lower$-PP^-$ and upper$-PP^+$ $\omega_T(q)$ branches near Γ ($\frac{q^2\times c^2}{\varepsilon_\infty} \to 0$) consist of the photon dispersion dictated by ε_s far-below the phonon resonance ($\omega \ll \omega_{TO}$) and of ω_{LO}, respectively. The corresponding asymptotic limits far away from Γ ($\frac{q^2\times c^2}{\varepsilon_\infty} \gg \omega_{LO}^2$) are ω_{TO} and the photon dispersion governed by ε_∞ far-beyond the phonon resonance ($\omega \gg \omega_{TO}$), respectively. The intermediary regime of strong phonon-photon coupling in the $\omega(q)$ diagram occurs when the horizontal phonon asymptote of the purely-mechanical TO mode away from Γ crosses the quasi vertical photon asymptote(s) near Γ. This gives rise to an anti-crossing of the lower$-PP^-$ and upper$-PP^+$ branches outside the *Reststhrahlen* ($\omega_{TO} - \omega_{LO}$) band, as shown in Fig. S1a. The latter band is thus forbidden for the PP coupling in volume. For more convenience the dimensionless parameter $y = \frac{q\times c}{\omega_0}$ substitutes for q in Fig. S1a, where ω_0 is arbitrarily taken identical to the ω_{TO} pulsation of the reference ZnSe crystal.

1.1-b. The SPP mode. By applying the Maxwell's equations to the evanescent wave propagating at the surface of a cubic crystal such as ZnSe, two independent sets of equations emerge for the magnetic ($\vec{B}$) and electric ($\vec{E}$) fields, corresponding to one transverse magnetic (TM) wave ($\vec{B}$ in-plane and $\perp$ $\vec{q}$) and one transverse electric (TE) wave ($\vec{E}$ in-plane and $\perp$ $\vec{q}$): obviously, the omitted $\vec{E}$ or $\vec{B}$ field in each case has both an out-of-plane component perpendicular to the surface as well as an in-plane one along $\vec{q}$. By writing the boundary conditions with air ($\varepsilon_r = 1$), it emerges that only the surface TM mode is likely to propagate (due to an absence of contrast at the air/dielectrics interface in what concerns the permeability).

The Maxwell's equations applied to the relevant TM waves on the air ($\varepsilon_r = 1$) and crystal [$\varepsilon_r(\omega)$] sides lead to

$$\alpha_{air} = \left(q^2 - \frac{\omega^2}{c^2}\right)^{\frac{1}{2}} \quad \text{and} \quad \alpha_{ZnSe} = \left(q^2 - \varepsilon_r(\omega)\times\frac{\omega^2}{c^2}\right)^{\frac{1}{2}}, \quad (5)$$

respectively, where the α −terms are the inverse of the characteristic evanescent lengths on each side of the air/crystal interface specified by the subscript. The boundary conditions derived from the Maxwell's equations for the TM wave at the air/crystal interface impose the continuity of the in-plane components of $\vec{E}$ together with the continuity of the displacement vector involving the out-of-plane component of $\vec{E}$, leading to $\varepsilon_r(\omega) = -\frac{\alpha_{ZnSe}}{\alpha_{air}}$. Both α −terms are positive (as needed to mimic the evanescent character of the surface TM wave away from the junction) so that $\varepsilon_r(\omega)$ is negative. Therefore the surface (TM) wave can propagate only within the *Reststrahlen* ($\omega_{TO} - \omega_{LO}$) band of the crystal – contrasting with the volume mode that can propagate only outside of it. The above continuity relation develops into the following dispersion for the surface (TM) mode,

$$\varepsilon_r(\omega) = \left(\frac{\omega^2}{q^2\times c^2} - 1\right)^{-1}, \quad (6)$$

leading to a second-order equation with solutions corresponding to the pulsation squared to the surface mode in their q-dependence given by

$$\omega_{surf.}^2(q) = \frac{q^2\times c^2}{2}\times\left\{\left(\frac{\omega_{LO}^2}{q^2\times c^2} + \varepsilon_\infty^{-1} + 1\right) \pm \left[\left(\frac{\omega_{LO}^2}{q^2\times c^2} + \varepsilon_\infty^{-1} + 1\right)^2 - \frac{4}{q^2\times c^2}\times\left(\omega_{LO}^2 + \frac{\omega_{TO}^2}{\varepsilon_\infty}\right)\right]^{\frac{1}{2}}\right\}. \quad (7)$$

The asymptotic solutions away from Γ ($\frac{\omega_{LO}^2}{q^2\times c^2} \to 0$) correspond to the photon dispersion scaled up that in vacuum by an effective refractive index (smaller than 1) of $\left(\frac{\varepsilon_\infty}{\varepsilon_\infty+1}\right)^{\frac{1}{2}}$ for the upper branch of the surface mode, and to the limit pulsation $\omega_{surf.} = \left\{\left(1+\frac{1}{\varepsilon_\infty}\right)^{-1} \times \left(\omega_{LO}^2 + \frac{\omega_{TO}^2}{\varepsilon_\infty}\right)\right\}^{\frac{1}{2}}$, falling within the *Reststrahlen* ($\omega_{TO} - \omega_{LO}$) band of the crystal (a more explicit form of ω_s with this respect is given below), for the lower branch. The asymptotic solutions near Γ ($\frac{\omega_{LO}^2}{q^2\times c^2} \gg \varepsilon_\infty^{-1} + 1$) are ω_{LO} for the upper branch and the photon dispersion scaled up that in vacuum by the effective refractive index (again smaller than 1) of $\left(\frac{\varepsilon_s}{\varepsilon_s+1}\right)^{\frac{1}{2}}$. The detailed y-dependencies of the two branches of the surface mode are shown in Fig. S1b. The (bimodal) surface mode is dispersive only in the (ω, y) −domain situated beyond the dispersion of light in vacuum ($\omega = c \times q = y \times \omega_0$), where the reported PP dispersions have no physical meaning. In the physically realistic portion of its $\omega(q)$ dispersion, the surface mode is nearly dispersionless, bound to ω_{surf}.

1.1-c. Raman cross sections of (TO, LO, surface) modes in their q-dependence. As we are basically interested in the Raman detection of the TO mode (covering both its purely-mechanical-TO and PP variants) it is useful to gain an insight into the TO Raman cross section in its q-dependence, to complete the available information on the $\omega_T(q)$ dispersions derived in the previous sub-Section. The procedure is extended to the LO mode and to the surface mode, for the sake of completeness – although these are of marginal interest in this work.

A theoretical insight into the q-dependent Raman cross sections of the three (TO, surface, LO) types of optical modes, sufficient to fix ideas, is obtained by capturing the divergence(s) behind the characteristic dispersions of such modes after proper weighting by a pre-factor involving the (ZnSe and MgSe) Faust-Henry coefficients, using the expression established in Ref. 22 (main text). Depending on the considered TO, surface or LO mode, the resonance term expresses as $Im\left\{-\frac{1}{\Delta(\omega,q)}\right\}$ by taking $\Delta(\omega,q)$ equal to $\varepsilon_r(\omega) - \frac{q^2\times\omega^2}{c^2}$, $\varepsilon_r(\omega) - \left(\frac{\omega^2}{q^2\times c^2} - 1\right)^{-1}$ or $\varepsilon_r(\omega)$, correspondingly.

A prerequisite for doing so is to confer an imaginary character on $\varepsilon_r(\omega)$. This is achieved by incorporating a friction force in the equation of motion related to the elementary bond stretching (u) behind an optical mode. The friction force is proportional to the velocity $\dot{u}$, and writes $(-\mu \times \gamma \times \dot{u})$ where μ is the reduced mass of the bond (Zn-Se in this case) and γ (expressed in cm^{-1}) is the so-called damping coefficient. In practice γ identifies with the full width at half maximum of the damped resonance for each relevant $\Delta(\omega,q)$ −term. Introducing the friction force leads to replace (ω^2) in the above expression of $\varepsilon_r(\omega)$ by $[\omega \times (\omega + j \times \gamma)]$ – being clear that $j^2 = -1$. The only needed input parameters to implement the calculations of the above imaginary terms are the ω_{TO} (205 cm^{-1} for ZnSe) and ω_{LO} (252 cm^{-1} for ZnSe) pulsations, accessible, *e.g.*, in the conventional Raman spectra taken in the backscattering geometry, along with the ε_∞ value (5.36 for ZnSe), accessible, *e.g.*, by far-infrared absorption. The as-calculated Raman efficiencies for the TO, surface and LO modes of ZnSe in their q-dependence are figured out via the thicknesses of the curves representing the $\omega(q)$ dispersions of these modes in Fig. S1 (the γ value was arbitrarily fixed to 1 cm^{-1} in all calculations).

We observe that in the relevant (ω, q) domain by using optical techniques (situated beyond the dispersion of light in vacuum $\omega = c \times q$), the surface and LO modes exhibit no dispersion and have stable Raman intensities. Accordingly a straightforward insight into the pulsation and the Raman efficiency of the surface mode can be obtained by focusing on large q values, as accessible in a conventional Raman experiment done in the backscattering geometry. In this case the dispersion of the surface mode takes the well-known asymptotic form $\varepsilon_r(\omega_{surf}) = -1$. This leads to

$$\omega_{surf.}^2 = \omega_{TO}^2 + \frac{\varepsilon_\infty}{\varepsilon_\infty+1} \times (\omega_{LO}^2 - \omega_{TO}^2), \quad (8)$$

(a variant of the expression of $\omega_{surf.}$ given above) making clear that the pulsation $\omega_{surf.}$ of the surface mode falls within the *Reststrahlen* $(\omega_{TO} - \omega_{LO})$ band. A crude insight into the Raman cross section of the surface mode can likewise be obtained by calculating $Im\left\{\frac{1}{\varepsilon_r(\omega)-1}\right\}$ – after proper weighting by the pre-factor involving the Faust-Henry coefficients.

In contrast, the Raman intensity of the VPP mode that propagates outside the *Reststrahlen* $(\omega_{TO} - \omega_{LO})$ band in the form of the lower−PP^- and upper−PP^+ sub-modes is strongly q-dependent, as apparent in Fig. S1a. Remarkably the Raman efficiency of each mode collapses in the photon-like regime. Intuitively this makes sense, as only the matter-like modes are likely to scatter light efficiently.

1.2. Raman scattering by the VPP mode(s) of ZnSe. The dedicated techniques to study the phonon dispersion throughout the first Brillouin zone $\left[0,\frac{\pi}{a}\right]$ – where a is the lattice constant – that encompasses all the q values needed to describe an actual lattice vibration, are inelastic neutron[2] (INS) and X-ray[3] (IXS) scattering, being implemented on national-size instruments (using a nuclear reactor and a synchrotron, correspondingly). However, optical techniques, as conveniently operated at the laboratory scale, are better suited for our use. Indeed, due to the quasi-vertical dispersion of light, the latter techniques naturally operate at small q values where the VPP modes propagate, and with unequalled spectral resolution (less than 1 cm^{-1} to compare with ~10 cm^{-1} in the case of INS/IXS). In particular, inelastic visible light scattering, commonly referred to as Raman scattering, is interesting because the addressed q value can be tuned to a certain extent by playing with the scattering angle θ between the wavevectors of the incident laser beam $(\vec{k}_i)$ and of the scattered light $(\vec{k}_s)$ inside the crystal. Such flexibility can be used to penetrate deep into the PP dispersion, depending on the dispersion of the refractive index around the used laser line (see below).

1.2-a. Comparative discussion of the backward and (near-)forward Raman insights. Like an INS or IXS experiment, a classical Raman experiment done in the backscattering geometry (schematically operated in 'reflection', *i.e.*, with $\vec{k}_i$ and $\vec{k}_s$ taken antiparallel, corresponding to $\theta \sim 180°$) fails to achieve the PP regime. Indeed, the wavevector $\vec{q} = \vec{k}_i - \vec{k}_s$ of the lattice vibration involved in the scattering process is maximum in this scattering geometry, roughly corresponding to $2 \times \vec{k}_i$ ($\vec{k}_i$ and $\vec{k}_s$ refer to close pulsations ω_i and ω_s in the visible spectral range, merely separated by the far-infrared pulsation of the lattice vibration involved in the inelastic scattering process, and thus have comparable magnitudes). Though this remains small at the scale of the Brillouin zone size, falling within the first few percent of it, the corresponding q value for the lattice vibration remains far away from the quasi vertical dispersion of a photon in the far-infrared spectral range. Therefore the TO mode detected in the backscattering geometry is deprived of electric field, being probed in the native purely-mechanical regime of the VPP mode.

The VPP regime can be accessed using Raman scattering by adopting the near-forward scattering geometry (schematically operating in 'transmission'). In this geometry $\vec{k}_i$ and $\vec{k}_s$ are (quasi) parallel, corresponding to $\theta \sim 0°$, so that $\vec{q}$ is minimum. In fact, the q_{min} value achieved by near-forward scattering is scaled down the q_{max} one addressed in the backscattering geometry by roughly two orders of magnitude, meaning that q_{min} is of the order of one per ten thousand of the Brillouin zone size. This falls close to the dispersion of a photon in the far-infrared spectral range. At this limit the photon-like electric field of a polar TO vibration is restored, and the latter excitation takes an actual VPP character.

The particular VPP mode probed by near-forward Raman scattering at a given scattering angle θ with a given laser line (ω_i) is identified by superimposing onto the VPP dispersion of the crystal the $\omega_s(q)$ dispersion scanned experimentally with the used scattering geometry, as dictated by the wavevector conservation law $(\vec{q} = \vec{k}_i - \vec{k}_s)$, leading to

$$q = c^{-1} \times \left\{n^2(\omega_i) \times \omega_i^2 + n^2(\omega_s) \times \omega_s^2 - 2 \times n(\omega_i) \times n(\omega_s) \times \omega_i \times \omega_s \times \cos\theta\right\}^{\frac{1}{2}}. \quad (9)$$

In this relation $n(\omega_i)$ and $n(\omega_s)$ are the refractive indexes of the crystal at the pulsations of the laser line and of the scattered light, respectively. The dispersion of the ZnSe refractive index, carefully measured throughout the visible spectral range by Peiris *et al.* using thin films (Ref. 37, main text), was found to obey the Sellmeier-type relation $n^2(\lambda) = A + B \times \lambda^2 \times (\lambda^2 - C)^{-1}$, where A, B and C are dimensionless constants being estimated as 4.049, 1.855 and 0.124, respectively, with λ given in the units of micrometers. The experimental Raman 'scan' lines derived for ZnSe on this basis for the blue (B, 488.0 nm), green (G, 514.5 nm), red (R, 632.8 nm) and near-infrared (IR, 785.0 nm) laser lines in the perfect forward scattering geometry ($\theta = 0°$) and for the finite scattering angle $\theta = 2°$ are superimposed onto the ZnSe VPP dispersion in Fig. S1a, after conversion to the relevant (y, ω_s) coordinates.

Note that the upper PP^+ branch cannot be accessed by Raman scattering. In contrast, the lower PP^- branch can be probed to a large extent. The dispersion of the ZnSe refractive index reduces with the energy, leading to a deeper penetration downward the PP^- dispersion towards Γ. In particular, the red laser line grants an access to the PP^- mode down to q values in the asymptotic photon-like regime, corresponding to quasi extinction of the Raman signal. Whichever laser line is used, as small departure from the perfect forward scattering as 2° suffices to recover the asymptotic purely-mechanical TO regime away from Γ.

1.2-b. The reference near-forward Raman study of ZnSe. Fig. S2 displays a representative series of near-forward Raman spectra ($\theta \sim 0°$) taken with ZnSe using the 488.0 nm (B) laser line at various near-normal incidences throughout parallel (110) crystal faces obtained by cleavage of a monocrystalline ZnSe ingot ($\sim$0.25 mm in thickness) with zincblende structure grown with the same method as the $Zn_{0.74}Mg_{0.26}Se$ mixed crystal studied in the main part of the manuscript. The used scattering geometry is TO-allowed (and LO-forbidden) and thus well-suited for the study of the VPP modes. Note that the purely-mechanical TO mode is visible besides the PP^- feature in each near-forward Raman spectrum. This is due to multi-reflection of the laser beam between the parallel faces of the transparent ZnSe crystal at normal incidence. With this, the near-forward PP^- Raman signal produced by the incident laser beam on its way forth to the top face of the crystal (detector side) superimposes on top of the backward Raman signal of its native purely-mechanical TO mode generated by the laser beam on its way back to the bottom face of the crystal (laser side) after reflection at the surface of the crystal (detector side), providing a useful reference. The multiple reflection of the laser beam is likewise responsible for the emergence of the theoretically forbidden LO modes (Ref. 9, main text).

As the scattering angle (θ) decreases, the TO mode engages its PP^- regime and develops as a pronounced shoulder on the low-pulsation tail of its native purely-mechanical TO mode, eventually showing up as a distinct feature that progressively shifts away from the latter reference. For each near-forward Raman spectrum, the exact scattering angle θ (specified besides each curve of Fig. S2) is estimated by identifying the relevant scan line which intercepts the PP^- dispersion at the detected pulsation. The latter was adjusted via a careful contour modeling of the bimodal (TO, PP^-) pattern using two symmetrical Lorentzian functions. One crucial parameter provided by the contour modeling is the damping (γ) of the PP^- mode (corresponding to the full width at half maximum of the Raman peak) in its θ −dependence (specified in Fig. S2). The basic trend is that γ increases as the scattering angle θ reduces. We have checked that no better insight into the θ −dependence of γ can be obtained by using the less energetic 514.5 nm (G) or 632.8 nm (R) laser lines. Indeed, with such laser lines the first-order PP^- Raman signal from the zone center suffers a dramatic decrease due to the basic ω^4 −dependence of the Raman efficiency, to the point that it becomes partly screened by the two-phonon Raman signal stemming from the edge of the Brillouin zone that emerges nearby, as earlier discussed in Ref. 23 (main text, see Fig. 7 therein).

The zone-center TO mode of ZnSe is nearly resonant with the two-phonon transverse-acoustical continua from the K and L zone-edges ($2A$) that emerge nearby on its low pulsation side (Ref. 31, main text). When the TO mode engages its PP^- regime and shifts towards low pulsation, the resonance conditions are better-matched which reinforces the Fano-type interference between the discrete (PP^-) and continuum ($2A$) states, eventually resulting in an anharmonic decomposition of the PP^-

mode into two zone-edge TA modes with opposite wavevectors (so as to fulfill the wavevector conservation law). This, we believe, is the main reason for the increased overdamping of the PP^{-} mode while shifting farther away from its native purely-mechanical TO mode (see also Ref. 8, p. 126, main text), with linewidth γ_0. In fact, the above described Fano-type interference is a common feature of ZnSe-based mixed crystals[4,5].

2. The $Zn_{0.74}M\gamma_{0.26}Se$ mixed crystal: structural, optical and basic vibrational properties

The crystal structure of the considered $Zn_{0.74}Mg_{0.26}Se$ single crystal together with the dispersion of its refractive indices in the visible, where the Raman scattering operates, are required prior to engaging the backward/forward Raman studies of its vibrational properties. As a preamble to the discussion of such properties we justify briefly why the composition of 26 at.%Mg was found most relevant for the current study.

2.1. Sample growth. The pure ZnSe and MgSe compounds crystallize in the zincblende[6] and rock-salt[7,8] (*i.e.*, NaCl-like, unstable at normal conditions, see also Ref. 31 main text) structures at ambient conditions, respectively. Therefore the $Zn_{1-x}Mg_xSe$ mixed crystal exhibits structural transitions when the composition changes. At Mg contents lower than 18 at.% and higher than 70 at.%, $Zn_{1-x}Mg_xSe$ adopts the parent-like zincblende[6] and rock-salt[9] structures, respectively. In the intermediary composition domain, the structure is wurtzite[6,7], of current interest. The highest achievable Mg content with the used variant of the Bridgmann method described in Ref. 6 is 63 at.%. The crystals with Mg contents larger than about 40 at.% are unstable in the air atmosphere. The 'pure' wurtzite structure is stable only in the restricted composition domain of 25-40 at.% Mg. Close to the zincblende-to-wurtzite transition (18-24 at.% Mg) the wurtzite-type crystals exhibit polytypes as the main or secondary phases[10]. As for the zincblende-type crystals, they usually contain twins.

The retained composition of 26±0.2 at.% Mg (see below) is the best compromise between high structural quality (absence of polytypes), minimization of the chemical and substitutional disorders (maxima at 50 at.% Mg) and sufficient incorporation of the light species to provide a distinct Raman signal (25 – 30 at.% is fine with this respect if we refer to $Zn_{1-x}Be_xSe$ and $ZnSe_{1-x}S_x$ – Refs. 22-26 main text). The composition gradient along the growth axis of the cylinder is negligible (less than 0.5 at.% Mg) for the considered height of sample.

2.2. X-ray diffraction measurements. The diffraction pattern obtained from a piece of the used $Zn_{0.74}Mg_{0.26}Se$ crystal for the Raman measurements finely ground into a powder by X-ray diffraction measurements done at the PSICHÉ beamline of the synchrotron SOLEIL using the 0.3738 Å radiation is shown in Fig. S3. It reveals the wurtzite structure of our sample. The X-ray diffraction lines compare in linewidth (several arcsec) with those of a (zincblende) $Zn_{1-x}Be_xSe$ crystal with small incorporation of the light (Be) cationic species (6 at.%) grown using the same method as the currently considered $Zn_{0.74}Mg_{0.26}Se$ crystal[11]. This testifies for the high structural quality of the latter crystal. Its composition was determined with an accuracy of ± 0.2 at.% Mg from the a (4.0500 Å) and c (6.6219 Å) lattice constants that were independently shown to vary linearly with the Mg content (using several techniques)[7].

2.3. Transmission ellipsometry measurements. To get to the bottom of the issue as to whether the structural anisotropy generates, or not, a significant linear birefringence $LB(\omega) = |n_0(\omega) - n_e(\omega)|$ in the visible spectral range (ω), we have implemented sensitive ellipsometry measurements at normal incidence in transmission throughout the $\vec{c}$ –containing faces of the small oriented $Zn_{0.74}Mg_{0.26}Se$ crystal. The transmission geometry is interesting because it provides a direct access to the Müller's (4×4) matrix that contains all information on the optical anisotropy of a crystal (see Ref. 36, main text). Of particular interest for our use are the $\widetilde{M}_{12}$ and $\widetilde{M}_{34}$ matrix elements shown in Fig. S4. Once divided by $\frac{2\pi\times d}{\lambda}$, where d and λ are the crystal length and the wavelength of the incident

light, respectively, they express the linear birefringence (LB) and the linear dichroism (LD), respectively, the two being connected via a Kramers-Krönig transformation (Ref. 36, main text).

The measurements were done as follows. The crystal was step-rotated (by 20°) around the direction of the incident light while maintaining a normal incidence onto the sample surface until the linear polarization ($\vec{e}_i$) of the incident light completes a revolution at the crystal surface. For each polarization angle $\alpha = (\vec{e}_i, \vec{c})$, the relevant ellipsometric parameters were measured throughout the visible spectral range, corresponding to the radial profiles in the compact-circular notation adopted for $\widetilde{M}_{12}$ and $\widetilde{M}_{34}$ in Fig. S4. Both matrix elements exhibit symmetrical patterns with respect to the $\vec{c}$ −crystal axis, demonstrating the optical anisotropy of the used crystal (otherwise the Müller's matrix would be diagonal). Remarkably, the radial profiles are quasi stable at each polarization angle α, indicating that the LB and LD values remain basically constant throughout the visible spectral range. In particular $\widetilde{M}_{12|\alpha=0°} > 0$, indicating that our $Zn_{0.74}Mg_{0.26}Se$ crystal is uniaxial positive ($n_e > n_O$ at any visible pulsation ω) – the corresponding ellipsoid of refractive indices is schematically represented in Fig. S5. However, the exact LB value, close to zero, cannot be estimated from the raw LB data (not shown) due to parasitic interferences created by multiple reflection of the incident light between the parallel faces of the transparent crystal. In contrast, the LD spectrum is well resolved – a representative spectrum taken at $\alpha = 0°$ corresponding to the best signal-to-noise ratio is shown in Fig. S4 (thin curve). It has been used to reconstruct analytically the difference spectrum $\delta[LB(\omega)] = LB(\omega) - LB(0)$ via a Kramers-Krönig transformation. The resulting $\delta[LB(\omega)]$ values, displayed in Fig. S4 (thick curve), hardly exceed 2×10^{-6} in magnitude throughout the visible spectral range. This is consistent with existing $Zn_{1-x}Mg_xSe$ data in the literature that indicate a stable LB value (in fact, a small one not exceeding 0.020 – Refs. 12 and 13) throughout the visible for Mg contents taken on both sides (~5 and ~45 at.%) of the current one (26 at.%). This constitutes an upper estimate for our crystal. We have checked that a uniform increase/decrease by ± 0.020 of the dispersion $n_{eff}(\lambda)$ of the effective refractive index of $Zn_{0.74}Mg_{0.26}Se$ throughout the visible spectral range (as measured by conventional ellipsometry with the non-oriented crystal) has strictly no impact on the detection of the PP modes by near-forward Raman scattering, whichever scattering angle (θ) or laser line (ω_i) is considered. In any case the change remains within the thickness of the (Raman) scan line, as already mentioned (see Methods, main text).

2.4. Backscattering Raman measurements. Selective Raman insights into the native A_1 and E_1 purely-mechanical TO's behind the PP's are obtained in the conventional backscattering geometry (schematically operating in reflection, $\theta \sim 180°$) with the red line (633.0 nm) of a HeNe laser at normal incidence onto the crystal faces with in-plane $\vec{c}$ −axis using crossed or parallel polarizations of the incident laser beam ($\vec{e}_i$) and of the scattered light ($\vec{e}_s$), as specified in Fig. S5. The used polarization setups are numbered in correspondence with the theoretical Raman selection rules schematically represented by the multi-lobe patterns, as calculated by using the relevant Raman tensors for a wurtzite-type crystal[14]. The unique A_1-like Raman tensor involves two distinct parameters (currently noted a and b) whereas the two Raman tensors referring to the E_1 symmetry are composed of the same unique parameter (labeled c hereafter). The double parametrization in the A_1 case introduces some ambiguity regarding the overall shape of the α-dependence of the Raman efficiency due to the A_1-like TO feature in crossed polarizations of the incident laser beam and of the scattered light. In fact the latter α-dependence takes the generic form $(b-a)^2 \cos^4 \alpha + 2a(b-a) \cos^2 \alpha + a^2$. Depending on the used a and b values, one may obtain significantly different contours for the α-dependence, corresponding either to ellipsoids oriented along or perpendicular to the $\vec{c}$-axis – with a shrinkage in the middle or not, or to a fully symmetrical four-petal pattern guided by both the $\vec{c}$-axis and its perpendicular. In our case the theoretical Raman selection rules were found consistent with the experimental ones provided $b = a/2$. As for the alternative α-dependencies of TO Raman efficiencies concerning the A_1-like feature detected by using parallel polarizations of the incident laser beam and of the scattered light, or the E_1-like one in any polarization geometry, these fit into similar four-petal

patterns, either guided by the $\vec{c}$-axis ($\cos^4\alpha$ −dependence) or tilted by 45° from it ($\sin^4\alpha$ −dependence), as shown in Fig. S5.

Not surprisingly the 'heavy' (Zn-Se) bonds vibrate at a lower pulsation (ω_{TO}~200 cm^{-1}) than the 'light' (Mg-Se) ones (ω_{TO}~275 cm^{-1}), resulting in a well-resolved bimodal $[1 \times (Zn-Se), 1 \times (Mg-Se)]$ TO pattern for $Zn_{0.74}Mg_{0.26}Se$. A more refined 3-mode percolation-type description including a 2-mode fine structuring of the high-pulsation mode, as observed with $Zn_{1-x}Be_xSe$ and $ZnSe_{1-x}S_x$ (Refs. 22-26, main text), is not needed in this case (supported by an *ab initio* study in the dilute limits in the next Section). In both the Zn-Se and Mg-Se spectral domains the 'soft−A_1' (Zn-Se: 200 cm^{-1}, Mg-Se: 270 cm^{-1}) and 'hard−E_1' (Zn-Se: 202 cm^{-1}, Mg-Se: 280 cm^{-1}) TO's vibrate at close pulsations. Note that the E_1 modes are broader than the A_1 ones in both spectral ranges (see, *e.g.*, $\gamma_1 < \gamma_1'$ in Fig. S5). The forbidden E_1 −type LO mode (Zn-Se: 230 cm^{-1}, Mg-Se: 310 cm^{-1}) apparent with the $\vec{c}$ −containing crystal face likewise emerges close to its allowed A_1 −like LO counterpart (Zn-Se: 229 cm^{-1}, Mg-Se: 301 cm^{-1}) detected at normal incidence onto the crystal face perpendicular to the $\vec{c}$ −axis. In view of this, we may well consider the $A_1 - E_1$ degeneracy, in a crude approximation. As apparent in Fig. S5, the degeneracy further extends to the high-pulsation component of the E_2 mode, *i.e.*, E_2^H, as predicted in the ZnSe (Ref. 30, main text) and MgSe (Ref. 31, main text) parent compounds and as also independently observed in the alternative ZnSe-based $Zn_{1-x}Cd_xSe$ mixed crystal[15]. We emphasize that the E_2 mode is a non polar one, thus of marginal interest regarding the study of the PP's.

In brief, our wurtzite-type $Zn_{0.74}Mg_{0.26}Se$ crystal actually exhibits a distinct bimodal (Zn-Se, Mg-Se) TO pattern, as required to support a pronounced S-like dispersion of the PP^{int} mode. However, the A_1 and E_1 $TO's$ due to a given bond appear to be quasi degenerate.

3. *Ab initio* insight into the native purely-mechanical TO modes behind the VPP's

In recent years we have introduced a so-called percolation scheme for the basic understanding of the purely-mechanical TO modes apparent in the Raman and far-infrared spectra of the $A_{1-x}B_x$-like semiconductor mixed crystals with zincblende and diamond structures (see, *e.g.*, Ref. 22, main text, and references therein). The percolation model distinguishes between the vibrations of the like bonds of a given species depending on whether they vibrate in their like environment or in the foreign environment (of the alternative bond), leading to a generic two-mode behavior per bond. In practice, the bimodal pattern per bond – referred to as the percolation doublet of a bond – is clearly resolved only for the bond formed with the substituent with the smaller covalent radius. This is because the latter has more freedom than the other substituent to adjust its position so as to best accommodate the local strain resulting from the contrast in the bond physical properties, with concomitant impact on its vibration pulsation, being more diversified. Altogether, this results in an apparent three-mode behavior for the mixed crystals with zincblende structure. Note that the bond formed with the small substituent, *i.e.*, usually the short one, is generally also the light and stiff one, so that the percolation doublet usually shows up at high pulsation. Besides, the short bond experiences a tensile strain in the presence of the long bond. Accordingly the percolation doublet of the light/stiff/short bond consists of parallel branches bent downward the parent mode in a 'pulsation vs. composition' plot. The above picture is valid as long as the vibration of the short bond is dispersionless, the mode of the pure crystal constituting a natural reference with this respect. It can be strongly distorted in case of a significant phonon dispersion (see, *e.g.*, Ref. 22, main text).

The currently considered $Zn_{0.74}Mg_{0.26}Se$ mixed crystal offers an occasion to work out a version of the percolation scheme for an anisotropic mixed crystal, *i.e.*, a wurtzite-type (hexagonal) one in this case, besides the existing zincblende- and diamond-type versions for the isotropic (cubic) type. Due to the structural anisotropy we expect that the above description of the triply-degenerate percolation-type TO modes behind the VPP's of the zincblende-type mixed crystals duplicates in the relevant A_1 and E_1 phonon symmetries of a wurtzite-type mixed crystal.

Independently of the structural anisotropy, $Zn_{1-x}Mg_xSe$ is further interesting in that it exhibits a quite unusual contrast in its bond physical properties. Surprisingly, the light (Mg) and heavy (Zn) substituents have similar covalent radii (in the range 1.25 – 1.30 Å), albeit the difference in atomic

masses is considerable (~63%). This suppresses any preferential ability for any substituent to accommodate the local strain due to the lattice mismatch ($\Delta a/a$~3.7 %, referring to the cubic MgSe and ZnSe crystals)[16]. With this respect, the two bonds have to be treated on equal footing. In particular the pulsation gaps between the sub-modes of the related percolation doublets are expected to be comparable.

In fact the two bonds of the considered $Zn_{0.74}Mg_{0.26}Se$ crystal exhibit a similar vibration pattern in the conventional Raman spectra taken in the backscattering geometry (Fig. S5), of the 1-bond→1-mode type, with no obvious sign of any percolation-type fine structuring. Now, the full width at half maximum of the Mg-Se Raman signal is roughly twice as large as the Zn-Se one, suggesting a possible 1-bond→2-mode percolation-type fine structuring for the Mg-Se signal, corresponding to a three-mode $[1 \times (Zn-Se), 2 \times (Mg-Se)]$ behavior in total for $Zn_{1-x}Mg_xSe$. As the purely-mechanical TO mode of the parent MgSe compound is quasi dispersionless throughout the Brillouin zone (whether taken in the zincblende or wurtzite structures – Ref. 32, main text), then, provided the Mg-Se percolation doublet actually exists, it should be mostly shaped by the local strain (Ref. 22, main text). This recollects with another disconcerting feature of the $Zn_{1-x}Mg_xSe$ mixed crystal that the bond likely to exhibit a percolation doublet (Mg-Se in this case) is the long one, and not the short one. The local strain is thus reversed with respect to the cases so far treated in the literature in that the percolation-type bond experiences a compressive strain, and not a tensile one. We anticipate a dramatic distortion with respect to the established versions of the percolation doublet, possibly different in the A_1 and E_1 symmetries.

3.1. ***Ab initio* method.** A wurtzite-type three-mode $[1 \times (Zn-Se), 2 \times (Mg-Se)]$ version of the percolation scheme for the purely-mechanical TO modes of $Zn_{1-x}Mg_xSe$ is derived on the basis of *ab initio* calculations of the Γ-projected TO density of states (BZC-TO-DOS) organized so as to address the two opposite impurity limits (x~0,1). Two prototypal percolation-type impurity motifs are considered, namely an isolated impurity and a short chain of two connected impurity bonds, immersed in large-replicated (64-atom) (Zn,Mg)Se supercells with wurtzite structure. The chain is positioned either along the $\vec{c}$-axis or perpendicular to it, so as to cover the A_1 and E_1 phonon symmetries for that particular anisotropic crystal motif. Owing to the large supercell size, each double-impurity chain or isolated-impurity motif is well separated from its counterpart in the next supercell under the supercell-imposed periodicity, and thus fairly represents the "isolated defect" situation. The calculations are done by using the SIESTA code[17] within the frozen phonon technique[18]. The long-range (macroscopic) electric field was not specially modelled in the calculation. Therefore the Γ-projected PhDOS assimilates with the purely-mechanical Raman TO modes accessed in the conventional backscattering geometry. The calculation setup was similar to that earlier used for calculations on the $ZnSe_{1-x}S_x$ system (Ref. 22, main text) in what regards the construction of norm-conserving pseudopotentials and the choice of basis functions. Specifically, the Trouiller-Martins pseudopotentials[19] have been constructed with the following atom shell occupation numbers, preceding (set in brackets, in Bohr units) corresponding cutoff radii: {Zn} $4s^2$(2.28) $4p^0$(2.28) $3d^{10}$(1.09) $4f^0$(1.50); {Se} $4s^2$(1.90) $4p^4$(2.00) $3d^{10}$(1.20) $4f^0$(1.50); {Mg} $3s^2$(2.18) $3p^0$(2.56) $3d^0$(2.56) $4f^0$(2.56). The basis functions were of the "double-zeta with polarization orbitals" quality. The "energy shift" parameter, used in the SIESTA code to control the confinement of the basis functions, was set to 0.02 Ry. The exchange-correlation was treated within the local density approximation. The k-sampling (for weighted summation of electron properties over the Brillouin zone) corresponded to the $2 \times 2 \times 2$ divisions along the hexagonal Brillouin zone of the supercell (with shifting), that was tested to yield sufficient accuracy in the calculation of forces. Frozen-phonon calculations on each supercell were preceded by unconstrained relaxation of the supercell size and interatomic distances.

3.2. ***Ab initio* results in the dilute limits ($x\sim0,1$).** An *ab initio* insight into the limit pulsations behind the presumed three-mode $[1 \times (Zn-Se), 2 \times (Mg-Se)]$ purely-mechanical TO Raman pattern of $Zn_{1-x}Mg_xSe$ at both ends (x~0,1) of the composition domain is given in Fig. S6.

At $x\sim1$ the most relevant supercell likely to provide a simultaneous access to the three limit purely-mechanical TO $[1 \times (Zn-Se), 2 \times (Mg-Se)]$ pulsations per symmetry (A_1 and E_1) is a large (64-atom) MgSe-like one with wurtzite structure containing one Zn atom. Basically, the Mg-Se bonds away from Zn provide a parent-like TO_{MgSe}^{E1} mode ($\sim295\ cm^{-1}$) located at a higher pulsation than the TO_{MgSe}^{A1} ($\sim270\ cm^{-1}$) one – marked by circles in Fig. S6, because the bond length is longer along the $\vec{c}$ −axis than perpendicular to it. The (A_1 and E_1) MgSe-like pulsations are different near Zn due to the Zn-induced local distortion of the MgSe-like lattice. A careful examination of the Γ-projected PhDOS per Mg atom reveals a localized E_1-like Mg-Se vibration near Zn at a lower pulsation than the bulk TO_{MgSe}^{E1} reference. The mode softening in presence of Zn is not surprising since the Zn-to-Mg substitution presumably generates a local strain of the tensile type, as already mentioned. The local mode in question refers to the in-phase vibration of the three in-plane Se atoms connected to Zn against the remaining two in-plane Mg atoms to which they are bonded. Altogether, this results in an apparent clockwise/counterclockwise in-plane circular Mg-displacement around Zn against the three Se ones bonded to Zn (a schematic view is given in the upper/right panel of Fig. S6). Such collective in-plane Mg-Se stretching around Zn in the real 3D crystal can be discussed at 1D in terms of the E_1-like Mg-Se stretching in presence of Zn, being then naturally referred to as the $MgSe/Zn^{E1}$ impurity mode in our percolation-type notation.

For the atom displacement along the $\vec{c}$ −axis, one may have well supposed that the $MgSe/Zn^{A1}$ impurity mode would likewise emerge beneath the bulk-like TO_{MgSe}^{A1} one, for the same reason. However, this is not so. In fact, in the anisotropic wurtzite structure the Zn-induced in-plane tensile strain appears to be compensated by a compressive out-of-plane strain. This is apparent in that the out-of-plane Mg_4Se tetrahedron unit situated immediately above Zn along the $\vec{c}$ −axis happens to be more compact than the three in-plane Se-centered tetrahedron units connected to Zn, as emphasized in Fig. S6 (refer to the lower inset on the right hand side). The combined effects of the tensile strain suffered by the in-plane Se atoms bonded to Zn and of the compressive strain applying to the out-of-plane Mg atoms bonded to the latter Se atoms result in an apparent compressive strain for the corresponding Mg-Se bonds along the $\vec{c}$ −axis. With this, the $MgSe/Zn^{A1}$ impurity mode somewhat counterintuitively emerges at a higher pulsation than the bulk TO_{MgSe}^{A1} mode.

Now, we consider $x\sim0$. The $ZnSe{:}\,1Mg^{A1}$ and $ZnSe{:}\,1Mg^{E1}$ impurity modes due to vibration of an isolated Mg atom in ZnSe along the $\vec{c}$ −axis and perpendicular to it provide the limit pulsations for the Mg-Se branch of each of the A_1- and E_1-like Mg-Se percolation doublets that refers to Mg-Se stretching in the foreign ZnSe-like environment. As expected, $ZnSe{:}\,1Mg^{A1}$ vibrates at a lower pulsation than $ZnSe{:}\,1Mg^{E1}$ owing to the larger bond length along the $\vec{c}$ −axis than perpendicular to it. An unusual feature is that both $ZnSe{:}\,1Mg^{A1}$ and $ZnSe{:}\,1Mg^{E1}$ impurity modes ($x\sim0$) fall above the (TO_{MgSe}^{A1} and TO_{MgSe}^{E1}) parent references ($x\sim1$), meaning that the Mg-Se percolation doublets bend upward the parent modes, and not downward as is in the usual case. Such apparent anomaly is due to the reversal of the local strain in $Zn_{1-x}Mg_xSe$. As the percolation-type bond is the long species (namely Mg-Se), and not the short one as in the usual case, it experiences a reduction in bond length when immersed in the foreign (ZnSe-like) environment. This strengthens the bond, with concomitant impact on the vibration pulsation, being correspondingly increased.

Additional insight into the impurity modes related to the remaining branch in each of the A_1- and E_1-like Mg-Se percolation doublets due to Mg-Se stretching in its own environment (MgSe-like) is needed to complete the picture. The relevant impurity motif in this case consists of a duo of neighboring Mg atoms in ZnSe, forming a short $Mg-Se-Mg$ chain of two connected Mg-Se bonds out of which one is taken along the $\vec{c}$ −axis. As expected, the stretching of the (long) Mg-Se along the $\vec{c}$ −axis (out-of-plane) provides a A_1 impurity mode ($ZnSe{:}\,2Mg^{A1}$, left panel of Fig. S6, lower motif) at a lower pulsation than its E_1 variant ($ZnSe{:}\,2Mg^{E1}$, upper/right motif in Fig. S6) corresponding to stretching of the short Mg-Se bonds perpendicular to the $\vec{c}$ −axis (in-plane). An independent pure−E_1 insight can be achieved by tilting the $Mg-Se-Mg$ chain in-plane and examining the Mg-Se stretching along the chain (in the spirit of the 1D percolation approach – upper/left motif in Fig. S6). As expected, the two E_1 insights are degenerate within less than 2 cm^{-1}.

Interestingly, both $(ZnSe{:}\,2Mg^{A1}, ZnSe{:}\,2Mg^{E1})$ duo-like impurity modes vibrate at lower pulsations than the $(ZnSe{:}\,1Mg^{A1}, ZnSe{:}\,1Mg^{E1})$ isolated-like ones. This relates to a basic feature of dilute bonds that they are longer when they are self-connected than when they are isolated – irrespectively of the nature (compressive or tensile) of the local strain, with concomitant impact on the Raman pulsations. This means that the ordering of the two percolation-type Mg-Se sub-branches is the same for both A_1 and E_1 symmetries at $x{\sim}0$, whereas it is opposite at $x{\sim}1$, in fact identical to $x{\sim}0$ for A_1 but opposite for E_1. This extrapolates into a disconcerting crossing of the two E_1 sub-branches at intermediary composition. As for the two A_1 ones, they remain quasi parallel throughout the composition domain (Fig. S6).

The *ab initio* phonon calculations done in both dilute limits ($x{\sim}0{,}1$) are further instructive in what regards the ZnSe-like vibrations. In none of the used ZnSe-like supercells containing diluted Mg-impurity motifs ($x{\sim}0$) was it possible to detect any significant splitting of the Γ-projected PhDOS per Zn atom, whether the atoms vibrate along (A_1) or perpendicular to (E_1) the $\vec{c}$ −axis, away (*i.e.*, in their like environment) or close to (*i.e.*, in the foreign environment of) the Mg impurities. This reveals that the Zn-Se Raman signal is sensitive neither to the symmetry (A_1 vs. E_1) nor to the local environment (like vs. foreign), and thus remains fully degenerate, *i.e.*, of the basic 1-bond→1-mode, throughout the composition domain. Generally this is consistent with experimental findings that the (A_1, E_1) ZnSe-like TO Raman lines detected in the backscattering geometry are much more narrow than their MgSe-like counterparts (Fig. S5).

3.3. Wurtzite-type version of the percolation scheme for $Zn_{1-x}Mg_xSe$. An overview of the three-mode percolation-type behavior $[1\times(Zn-Se), 2\times(Mg-Se)]$ in the purely-mechanical TO regime of the $Zn_{1-x}Mg_xSe$ mixed crystal with wurtzite structure, extrapolated from the *ab initio* insights in the relevant parent (large/open symbols) and impurity (small/filled symbols) modes in the Mg-dilute ($x{\sim}0$) and -parent ($x{\sim}1$) limits is shown in Fig. S6 (main panel). The composition dependence of the three TO pulsations is inferred merely by connecting via straight (dashed) lines the limit *ab initio* pulsations due to a given oscillator in a given symmetry (A_1 or E_1) at the two ends of the composition domain. The fractions of individual oscillators (specified within square brackets), estimated by assuming a random Zn↔Mg substitution and a sensitivity of the Mg-Se vibrations to its first-neighbor environment (as is the case for most re-examined mixed crystals so far), complete the picture.

At any composition x the pulsation gap within the like sub-modes of a given (A_1 or E_1) Mg-Se percolation doublet hardly exceeds the natural damping $({\sim}10\,cm^{-1})$ of the MgSe-like purely-mechanical TO Raman feature apparent in the $Zn_{0.74}Mg_{0.26}Se$ Raman spectra (Fig. S5). Accordingly for subsequent use in the main part of the manuscript we may as well consider that the two sub-oscillators behind the Mg-Se percolation doublet revealed by the *ab initio* calculations merge into one single mode that attracts the whole available MgSe-like oscillator strength at the considered composition, in any symmetry (A_1 or E_1).

4. Near-forward Raman studies of the satellite PP^- and PP^+ modes

This Section. relates to the VPP modes originating from the purely-mechanical TO modes described in the previous Section. A theoretical insight into the $\omega(q)$ dispersion and Raman efficiencies of the VPP's (Sec. 4.1) is useful to support the discussion of alloy-related PP^{int} modes in the main text, and also to introduce the forecoming discussion of its satellite parent-like PP^- (Sec. 4.2) and PP^+ (Sec. 4.3) features, notably in their dependence on the used laser excitation (Sec. 4.3).

4.1. Theoretical insight into the VPP dispersion and related Raman intensities. In the conventional Raman spectra of $Zn_{0.74}Mg_{0.26}Se$ taken in the backscattering geometry (Fig. S5) the purely-mechanical TO modes exhibit a basic two-mode $[1\times(Zn-Se), 1\times(Mg-Se)]$ behavior with A_1-E_1 quasi degeneracy. Accordingly a classical two-oscillator form is taken for $\varepsilon_r(\omega)$, in which each (A_1, E_1) −degenerate mode is represented by a Lorentzian oscillator with a damped resonance at the relevant ω_{TO} pulsation with an oscillator strength (corresponding to the jump $\varepsilon_s-\varepsilon_\infty$ in the dielectric constant of the crystal caused by the related phonon resonance) scaling as the bond fraction. In doing

so we refer more specifically to the best-resolved TO features of the A_1 type (the full width at half maximum of the A_1 lines is at least one and a half times smaller than their E_1 counterparts). The electronic contribution (ε_∞) to $\varepsilon_r(\omega)$ is assumed to vary linearly between the parent values. Remaining input parameters related to the parent systems (the TO and LO pulsations and the high-pulsation relative dielectric constant ε_∞ far away from the phonon resonance) are taken from Ref. 32 (main text). The resulting (A_1 −like) Raman cross section for the three [ZnSe-like PP^-, (Zn-Se,Mg-Se)-like PP^{int}, MgSe-like PP^+] modes of $Zn_{0.74}Mg_{0.26}Se$ in its (ω, q) −dependence is displayed in Fig. 1a. The thickness of each PP branch indicates the Raman efficiency. For more convenience, we substitute for q the dimensionless parameter $y = q \cdot c \cdot \omega_0^{-1}$, where c is the speed of light in vacuum and ω_0 arbitrarily refers to the TO pulsation of ZnSe.

Strictly speaking, the VPP dispersion reported in the main text and calculated as indicated in the Methods Section (main text) is only valid for the ordinary polariton of A_1-type – whose polarization and wavevector ($\vec{q}$) are parallel to the $\vec{c}$ −crystal axis. Now, owing to the quasi-degeneracy of the native purely-mechanical A_1 and E_1 TO's behind the phonon-polaritons and to the apparent lack of linear birefringence (in the ellipsometry data), such A_1-type dispersion is presumably also valid for the E_1 phonon-polariton, that also propagates in the optically isotropic plane but with polarization perpendicular to the $\vec{c}$ −crystal axis. In fact, only the latter two types of phonon-polaritons are likely to be addressed in the perfect forward scattering geometry ($\theta = 0°$) at normal incidence/detection onto/from crystal faces with in-plane $\vec{c}$ −axis. However, the latter geometry is merely theoretical. Due to the finite numerical apertures of the used optics to focus the incident laser beam at the rear of the sample and to collect the scattered light from its front surface, a continuous distribution of scattering angles necessarily comes into play (see Sec. 4.3), and, along with it, finite scattering angles ($\theta \neq 0$). In this case the extraordinary polariton departs from the pure A_1 −type and acquires a mixed (A_1, E_1) −character. Its dispersion is then governed by an effective relative dielectric function $\varepsilon_{r,\psi}(\omega)$ involving the A_1 − and E_1 −like ones [commonly referred to as $\varepsilon_{r,\parallel}(\omega)$ and $\varepsilon_{r,\perp}(\omega)$, correspondingly] with relative weights depending on the angle ψ between $\vec{q}$ and the $\vec{c}$ −crystal axis. In fact, the angle ψ virtually spans all possible values between 0° and 90° (the surface mode, characterized by $\vec{q} \perp \vec{k}_i$ − referring to $\psi = 90°$ in the present case, was detected by near-forward Raman scattering at normal incidence onto $Zn_{0.67}Be_{0.33}Se$ on top of the nominal volume polariton for which $\vec{q} \parallel \vec{k}_i$ – thus referring to $\psi = 0°$ – see Ref. 22 – main text), corresponding to the pure A_1 and E_1 symmetries, respectively. We have checked (not shown) that, for the currently considered $Zn_{0.74}Mg_{0.26}Se$ mixed crystal, the resulting ψ −dependent dispersion for the extraordinary polariton, given by $\varepsilon_{r,\psi}(\omega) = q^2 \cdot \omega^2 \cdot c^{-2}$ (the developed expression is given, *e.g.*, by equation. (4-53) in Ref. 8 – main text) does not significantly deviate from the basic A_1 −like one displayed in Fig. 1a, at any angle ψ. The latter version of the VPP dispersion is thus sufficient and appropriate for our subsequent use.

An idea how far it is possible to penetrate downward the S-like dispersion of the PP^{int} mode towards Γ by near-forward ($\theta \sim 0°$) Raman scattering with the available near-infrared (NIR, 785.0 nm), red (R, 632.8 nm), green (G, 514.5 nm) and blue (B, 488.0 nm) laser lines spanning the visible spectral range is obtained by superimposing onto the theoretical VPP dispersion (Fig. 1a) the corresponding experimental 'scan' lines (dotted curves) derived from the wavevector conservation law $\vec{q} = \vec{k}_i - \vec{k}_s$ that governs the Raman scattering in the ideal case of a perfect forward scattering (θ=0°). In this geometry $\vec{k}_i$ and $\vec{k}_s$ are (nearly) parallel and in the same sense, so that the addressed q value is minimal, given by $q_{min} = c^{-1} \cdot |n_{eff}(\omega_i) \cdot \omega_i - n_{eff}(\omega_s) \cdot \omega_s|$, where ω_i and ω_s refer to the pulsations of the incident laser beam and of the scattered light, respectively. In the ellipsometry data, $n_{eff}(\omega)$ reduces with ω, and its derivative as well. Accordingly, the Stokes Raman scattering ($\omega_i > \omega_s$, adopted in this work) is preferred to the anti-Stokes one ($\omega_i < \omega_s$) in view to achieve a smaller q_{min} value. Further, q_{min} decreases by using less energetic laser lines. In fact the G and B laser lines hardly suffice to cover the PP^{int} collapse regime of $Zn_{0.74}Mg_{0.26}Se$ in full down to the photon-like extinction (I_c) and fall short of accessing the PP^{int} reinforcement regime of main interest (beneath I_c). As for the NIR laser line, it suffers from the ω^4-collapse of the Raman efficiency, which dramatically

impacts the signal-to-noise ratio in the case of $Zn_{0.74}Mg_{0.26}Se$ (aggravated by the relative emergence of the second-order Raman signal, rendering the understanding of the first-order VPP one a more complicated issue). Altogether this points towards the R laser line as the most relevant one to probe the S-like PP^{int} dispersion of $Zn_{0.74}Mg_{0.26}Se$ in the reinforcement regime.

The minimal achievable pulsation for the PP^{int} mode when using the R laser line (ω_{min}~258 cm^{-1}) – obtained in the perfect forward scattering geometry ($\theta = 0°$) – actually falls below ω_c ($\sim 265\ cm^{-1}$), but very close to it (Fig. 1a). This means that only the early stage of the PP^{int} reinforcement regime situated immediately underneath I_c is likely to be probed experimentally. Apparently we are not in a position to push the exploration of the S-like dispersion of the PP^{int} mode towards Γ beyond the limit already achieved with $Zn_{1-x}Be_xSe$ and $ZnSe_{1-x}S_x$ (Refs. 22-26 main text).

4.2. A "fractional" access to the PP^- mode. Remarkably, when shifting from the $X(ZZ)X$ pure$-A_1$ geometry (Fig. 1b) to the alternative $X(YY)X$ pure$-A_1$ one or to the $X(ZY)X$ pure$-E_1$ one, the sharp PP_{TO}^{int} feature disappears (Fig. S7). The reason in the $X(YY)X$ geometry is that the A_1 modes are marginally activated (refer to the tightening of the A_1 $-$like bi-lobe TO pattern when using parallel polarizations perpendicular to the $\vec{c}$ $-$axis), and moreover screened by the dominant contribution from the spurious E_2^H modes, that show up strongly in this specific geometry though they are theoretically forbidden. In the E_1 $-$like $X(YY)X$ geometry, the PP_{TO}^{int} is replaced by an overdamped band. The dramatic 'sharp→overdamped' transition may seem disconcerting owing to the quasi $A_1 - E_1$ degeneracy. Its explanation is a pure matter of Euclidian geometry as ultimately concerned with the intersection of two (quasi) horizontal $\omega(q)$ curves (the lower$-A_1$ and upper$-E_1$ S-like PP_{TO}^{int} dispersions) near a common singularity SI (standing for the photon-like extinction at I_c) which separates two distinct regimes of the PP^{int} mode (the reinforcement – R and collapse – C ones) by an oblique (straight) curve (standing for the Raman 'scan' line achieved with the red laser excitation) – as sketched out in Fig. S7. The oblique curve crosses the two parallels on both sides of SI, *i.e.*, in different regimes, with a dramatic impact on the PP^{int} feature showing up as a sharp (R regime) or overdamped (C regime) feature.

Interestingly, in the E_1 $-$like $X(YY)X$ geometry the disappearance of the PP_{TO}^{int} mode goes with the emergence of the ZnSe-like PP^- mode showing up as a distinct feature on the low-pulsation tail of the minor purely-mechanical TO_{Zn-Se}^{E1} mode. This vibrates at a pulsation (~192 cm^{-1}) by far exceeding the theoretical value (~150 cm^{-1}, refer to the crossing between the PP^- dispersion and the Raman 'scan' line at θ~0.55° in Fig. 1a). The shift can be explained only if the detected PP_{TO}^- mode carries a fraction of the available Zn-Se oscillator strength, and not the whole of it. This suggests a fine structuring of the native TO_{Zn-Se}^{E1} mode, which must not be surprising in a disordered system such as a mixed crystal. A close examination of the corresponding $X(ZZ)X$ A_1 $-$like near-forward Raman spectrum (Fig. 1a) further reveals its presence as a minor shoulder on the low-pulsation side of the dominant TO_{Zn-Se}^{A1} mode.

As apparent in Fig. 1a, the PP^- feature disappears when adopting the $X(YZ)X$ E_1 $-$like scattering geometry in which the laser beam propagates as the ordinary wave. This must not be surprising since the upward-tilted scan line that presumably applies in the latter geometry (refer to the curved arrow in Fig. 1a) fails to cross the PP^- dispersion. In contrast, the nominal scan line (at the departure of the curved arrow) valid for both $X(ZY)X$ and $X(ZZ)X$ geometries does actually cross the latter dispersion. However, the crossing occurs at a much lower pulsation ($\sim 130\ cm^{-1}$) than observed experimentally ($\sim 190\ cm^{-1}$), *i.e.*, deep into the photon-like regime of PP^- where the latter mode is not likely to scatter light efficiently (refer to the thickness of the PP^- curve at the crossing point with the scan line). The disconcerting emergence of a strong PP^- Raman signal close to its native TO_{Zn-Se} mode in the $X(ZY)X$ pure$-E_1$ geometry can be explained only if the observed PP^-feature does not carry the full amount of ZnSe-like oscillator strength nominally awarded to this mode (we recall that this scales as the bond fraction), but only a (small) part of it. This comes to consider a fine structure behind the native TO_{Zn-Se} mode, the result of natural fluctuations in the crystal composition at the local scale in case of a random atom substitution. In fact, the existence of such 'fractional' ZnSe-like PP^- features

has already been established on a similar basis with the zincblende-type $Zn_{0.47}Be_{0.53}Se$ and $ZnSe_{1-x}S_x$ (x=0.32, 0.22) mixed crystals (Refs. 23 and 24, main text). In the cited works the individual sub-modes from the fine structure were considered to engage their phonon-polariton regime separately, due to more or less pronounced anharmonic coupling with quasi resonant two-phonon zone-edge acoustical bands nearby ($2A$) that are well-known to interfere strongly with the zone-center TO Raman mode in ZnSe-based systems, as already mentioned (Sec. 1.2b).

A fair estimate of the actual amount of oscillator strength awarded to the observed PP^- mode is obtained by examining how the PP^- depends both on the laser excitation at ultimately small scattering angle, and on the scattering angle for a given laser excitation (*i.e.*, G, 514.5 nm in this case, see below). A representative series of near-forward Raman spectra taken by polarizing the laser beam parallel to the $\vec{c}$ −crystal axis ($\vec{e}_i \parallel \vec{c}$) and without analyzing the scattered light – so as to allow for the simultaneous recording of both the (A_1 −like) PP^{int} and (E_1 −like) PP^- features is displayed in Fig. S8a.

By referring to the pulsation of the PP^{int} mode detected with the red (R, 632.8 nm) laser line, the θ value is estimated at $\theta \sim 0.550°$, corresponding to the early stage of the PP^{int} reinforcement regime (see main text). The scattering angle outside the crystal (obtained via the effective refractive index measured by ellipsometry – see Fig. 2a – at the pulsation of the red laser line, *i.e.*, n_{eff}~2.425) is θ_{ext}~1.33°. The corresponding θ values inside the crystal for the near-infrared (IR, 785.0 nm, n_{eff}~2.380), green (G, 514.5 nm, n_{eff}~2.496) and blue (B, 488.0 nm, n_{eff}~2.526) laser lines are $\sim(0.560°, 0.535°, 0.528°)$, respectively. The $PP^{int.}$ mode apparent with the NIR laser line is consistent with the above θ estimate. As for the B and G laser lines, the corresponding θ values probe the PP^{int} mode in its collapse regime, where the mode remains weak and overdamped, the reason why it does not show up distinctly in the related near-forward Raman spectra.

The θ estimates likewise provide theoretical PP^-Raman lines near the experimental pulsations when using the B, G and R laser lines provided the effective amounts of oscillator strength awarded to this mode represents only $\sim 12\%$ of the available ZnSe-like oscillator strength (scaled down the parent value by the bond fraction). In this case the PP^- mode is also expected to show up with the NIR laser line but shifted well beneath ($\sim 20\ cm^{-1}$) the native TO_{Zn-Se} mode, where it presumably interferes destructively with the two-phonon acoustical bands (2-TA) nearby (see Sec. 1.2b).

Note the existence of an overdamped band, labelled X, at $\sim 20\ cm^{-1}$ below the main TO_{Zn-Se} mode in the near-forward Raman spectra taken with the B and G laser lines. By reducing the laser energy (from B to G) at a given θ angle (~0.53°) the X band shifts downward, whereas upward when θ increases using a given laser line (G). Altogether, this is consistent with an assignment of the X band in terms of an additional PP^- −like feature, further supporting our view that the native TO_{Zn-Se} mode is finely structured. From now on the X and PP^- features are labelled as PP^-_{low} and PP^-_{high}, respectively. The experimental PP^-_{low} and PP^-_{high} pulsations detected at ultimately small scattering angle ($\sim$0.53°) with the B and G laser lines are consistent with theses modes being awarded as much as $\sim$72% and $\sim$12% of the available ZnSe-like oscillator strength, respectively. Such sharing of oscillator strength is independently validated by the θ-dependence of both pulsations observed with the G laser lines when θ varies in the range 0.53° – 0.68° (Fig. S8b).

The overdamping of the PP^-_{low} mode, contrasting with the sharpness of the PP^-_{high} one, reflects a natural trend observed with the PP^- mode of the pure ZnSe crystal that it becomes increasingly damped on shifting away from its native TO_{Zn-Se} mode, due to the reinforced anharmonic coupling with the $2A$ continuum nearby (see Sec. 1.2b). By using less energetic laser lines (R and NIR) the shift enlarges and thus also the overdamping, leading to disappearance of the PP^-_{low} mode.

The above discussion is supported by a contour modeling of the ($PP^-_{low}, PP^-_{high}, PP^{int}$) Raman lineshapes (Fig. S8b). This was achieved by using the same set of input parameters taken from Ref. 32 (main text), with the fractions of ZnSe-like oscillator strengths specified above (Sec. 3.3). Three types of theoretical $Zn_{0.74}Mg_{0.26}Se$ Raman lineshapes are calculated:

(i) The reference purely-mechanical TO ones (thick lines) derived via $Im\{\varepsilon(\omega,x)\}$ using a crude 2-mode $[1\times(Zn-Se),1\times(Mg-Se)]$ description in which each mode is awarded its nominal amount of oscillator strength (scaling as the bond fraction);

(ii) The corresponding VPP ones (thin lines) obtained via $Im\left\{-\frac{1}{\varepsilon_r(\omega)-\frac{q^2\times\omega^2}{c^2}}\right\}$ at the relevant scattering angles, giving access to the $PP^{int.}$ Raman lineshapes;

(iii) The VPP ones calculated along the same line as above and using the same scattering angles but on the basis of a "truncated" 2-mode description in which the Zn-Se mode is awarded only a fraction of its nominal oscillator strength, *i.e.*, either 72% or 12% of it, depending on whether the PP^-_{low} or PP^-_{high} feature is addressed, respectively.

In each case, the relevant resonance (imaginary) term is weighted by the pre-factor involving the (ZnSe and MgSe) Faust-Henry coefficients, using the generic formula for the Raman cross section established in Ref. 22 (main text). A minimal phonon damping ($1\ cm^{-1}$) is used for clarity. No change in damping is taken into account, neither its drastic decrease when crossing the extinction of the PP^{int} mode and passing from the collapse to the reinforcement regime, nor its progressive increase for the PP^-_{low} and PP^-_{high} modes when they penetrate deeper their phonon-polariton regime and interfere destructively with the $2A$ continuum.

In brief, the native purely-mechanical TO_{Zn-Se} mode behind PP^- appears to be finely structured due to the alloy disorder. Interestingly the sub-modes engage separately the PP regime presumably due to more or less pronounced interferences with the $2A$ continua nearby. This eventually leads to multiple 'fractional-like' PP^-sub-features.

4.3. The asymmetrical PP^+ feature. While the E_1 −like PP^{int}_{LO} mode detected with the red (633.0 nm) laser line in the $X(YZ)X$ geometry is sharp and symmetrical (the full width at half maximum is less than 10 cm^{-1}), the accompanying PP^+_{LO} mode exhibits a pronounced asymmetry on its high-pulsation side extending over ~50 cm^{-1} (Fig. 2b).

The sharpness of the $PP^{int.}$ mode was discussed in Ref. 24 as being due to a coupling between the sub-modes (due to inherent fluctuations in the local composition in a mixed crystal) forming the fine structure of the native purely-mechanical TO phonon behind the PP^{int} mode. The coupling is mediated by the PP-like electric field $\vec{E}$ once this has developed into an actual macroscopic one, on entry in the photon-like regime. The $\vec{E}$ −coupling channels the available oscillator strength towards the lower sub-mode of the series[22], eventually leading to the formation of a sharp and giant $PP^{int.}$ feature.

The asymmetry of the PP^+_{LO}mode reflects a distribution of scattering angles due to the finite numerical apertures of the rear and front lenses used to focus the incident laser beam at the rear of the crystal (producing a finite distribution of incidence angles, as sketched out in Fig. S9) and to collect the scattered light (selecting a finite distribution of emission angles, see Methods). The maximum scattering angle outside the crystal hardly reaches 4.2°. By taking ~2.40 for the effective refractive index of the crystal in the red spectral range (measured by ellipsometry), the scattering angle inside the sample falls below ~1.75° when using the red laser line. The detected Raman signal taken is thus due to a continuous series of scattering angles between the minimal value of 0.55° (that was found relevant to model A_1 −like $X(ZZ)X$ Raman signal using an unique scattering angle) and the maximum value of 1.75°.

An additional ingredient is required to achieve a crude contour modeling of the combined PP^{int} and PP^+ features seen in the E_1 −like $X(YZ)X$ spectra recorded with the red laser line, namely the dispersion of the (ordinary) refractive index around the latter laser line after distortion by the resonance process. We must admit that we have no idea of the exact shape of the distorted dispersion. Based on experimental findings we are only in a position to state that the resonance process has lead to a local inversion of the dispersion (see main text), a positive one (n reduces with ω) if we refer to the ellipsometry data, eventually reversed into a negative one by the resonance process. For simplicity

the distorted $n_0(\omega)$ dispersion is taken linear. In addition we assume that the refractive index at the pulsation of the red laser line is the same whether using the nominal or distorted dispersions of n_0. The only adjustable parameter is the slope of the distorted dispersion then. This was adjusted so that the theoretical q-dependent VPP Raman cross section (RCS) of $Zn_{0.74}Mg_{0.26}Se$, that contains the information on the (distorted) dispersion of n_0 via the relation between q and θ dictated by the wavector conservation law (see Sec. 1.2a), generates PP_{LO}^{int} and PP_{LO}^{+} features with pulsations close to the experimental values. As a crude approximation of the multi-VPP Raman cross section, we used the generic formula,

$$RCS(\omega) \sim \int_{\theta=0.55°}^{1.75°} Im\left\{-\frac{1}{\Delta(\omega,x,\theta)}\right\} \times \sin\theta \times d\theta, \quad (10)$$

in which $\omega = \omega_i - \omega_s$ refers to the Stokes shift. $\Delta(\omega,x,\theta)$ is equal to $= \varepsilon_r(\omega) - \frac{q^2 \times \omega^2}{c^2}$ with q depending on θ as specified above, and $\varepsilon_r(\omega)$ is the two-oscillator $[1 \times (Zn - Se), 1 \times (Mg - Se)]$ relative dielectric function of the considered $Zn_{0.74}Mg_{0.26}Se$ mixed crystal. In fact, we used the proper expression of RCS given in Ref. 22 (main text), with the weighting factor including the (ZnSe and MgSe) Faust-Henry coefficients. In practice the above integral was calculated as a Riemann sum using a small angular (θ) step of 0.025°.

The best agreement between the theoretical and experimental PP_{LO}^{int} and PP_{LO}^{+} pulsations is achieved by taking a slope for the linearly distorted dispersion twice as large as the nominal one (also considered to be linear locally), with opposite sign. By doing so the theoretical PP_{LO}^{int} and PP_{LO}^{+} pulsations slightly underestimate (by ~12 cm^{-1}) and overestimate (by ~6 cm^{-1}) the experimental values, respectively. The corresponding envelope Raman signal (thick curve) representing the sum of the individual ($PP_{LO}^{int}, PP_{LO}^{+}$) Raman signals weighted by $\sin\theta$ produced each step-increase in the scattering angle of 0.025° between 0.55° and 1.75° (thin curves, ranked from low- to high-pulsation in the sense of increasing angle) is shown in Fig. S9. As naïve as it is, our theoretical approach is rather successful at conciliating the sharpness of the PP_{LO}^{int} Raman line along with the large high-pulsation asymmetry of the PP_{LO}^{+} one. Moreover, the experimental $PP_{LO}^{int}/PP_{LO}^{+}$ intensity ratio is fairly well reproduced theoretically.

In fact the large asymmetry of the PP_{LO}^{+} mode arises because the relatively small (resp. large) Raman intensity of the individual PP_{LO}^{+} modes at small (resp. large) scattering angles, that emerge at small (resp. large) pulsations, is somehow compensated by the correspondingly large (resp. small) density of states of the PP^{+} dispersion. The two effects (PP^{+} Raman intensity, PP^{+} density of states) more or less neutralize each other, so that the contributions to the PP^{+} Raman signal resulting from small and large scattering angles are comparable. There is a slight advantage to the low-pulsation modes though, which suffices to confer a pronounced asymmetry on the high-pulsation side of the envelope PP_{LO}^{+} Raman signal. Such 'balance' between 'PP^{+} Raman intensity' and 'PP^{+} density of states' is absent for the PP^{int} mode. Indeed the elementary PP^{int} modes corresponding to small (resp. large) scattering angles, that also occur at low pulsation, benefit (resp. suffer) at the same time from a strong (resp. weak) Raman efficiency and from a large (resp. small) density of states. Therefore the low-pulsation modes provide a dominant and quasi-uniform contribution to the envelope PP_{LO}^{int} signal, thus showing up as a quasi symmetrical feature.

In the above description we neglect the vibrational and optical anisotropies of our wurtzite-type crystal and consider a symmetry of revolution around the direction of the laser beam that propagates perpendicularly to the $\vec{c}$-crystal axis. In fact the symmetry of revolution is relevant only in case of a perfect forward Raman experiment done along the $\vec{c}$-axis. However, our naïve approach can be justified, in a very crude approximation, due, on the one hand, to the quasi degeneracy of the phonon modes in the A_1 and E_1 symmetries, and, on the other hand, to the negligible linear birefringence revealed by ellipsometry. A more rigorous treatment would require to take into account the absence of symmetry of revolution and to model the Raman signal stemming from a particular solid angle with respect to the $\vec{c}$-crystal axis using well-defined polarizations of the laser beam and of the scattered light, in the spirit of the approach recently developed by Irmer *et al.* (Ref. 11, main text).

In fact, the inversion (slope doubled, with opposite sign) of the refractive index dispersion (ideally taken as linear) near ω_i can be mimicked by adding to the measured $n_{eff}^2(\omega)$ dispersion one unique (to keep the procedure as simple as possible) damped Lorentz oscillator. This introduces a complex refractive index $n^*(\omega) = n(\omega) + jk(\omega)$, given by

$$n^{*2}(\omega) = n_{eff}^2(\omega) + \mathrm{A} \cdot \frac{\omega_{Res}^2}{\omega_{Res}^2 - \omega^2 - 2j\gamma\omega} . \quad (11)$$

In this expression the fraction represents a normalized resonance at pulsation ω_{Res}, being damped over the pulsation domain 2γ [corresponding to the width at half maximum of the $k(\omega)$ peak]. The so-called oscillator strength A fixes the magnitude of the damped resonance. The position ω_{Res} of the resonance is observed experimentally; it coincides with the sudden extinction of the enhanced PP Raman signal. For example, in the near-forward Raman spectra recorded with the red laser line the resonance occurs at $\omega_{Res} \sim \omega_i - 840$ cm^{-1} (Fig. 2b). The damping term is fixed at $\gamma = \omega_i - \omega_{Res}$, *i.e.*, the minimum value that guarantees an inversion of the refractive index $n(\omega)$ dispersion throughout the studied $\omega_{Res} \leq \omega_s \leq \omega_i$ spectral domain. This leaves A as the only adjustable parameter. The inversion of the refractive index dispersion shown in Fig. 2b (main text) was obtained by taking A~0.03. By way of comparison the A-estimate represents ~1% of the oscillator strength ($\varepsilon_s - \varepsilon_\infty$) of the optical phonon of ZnSe.

While the estimated amount of oscillator strength A stays beyond the ellipsometry detection threshold with the used ellipsometry setup (Methods Section, main text) by a factor four, it remains of the same order. More precisely, we have checked that once A is divided by four, the singularity related to the damped Lorentz oscillator does not emerge from the measured noise associated with the actual physical data measured by ellipsometry (*i.e.*, the sine and cosine functions of the depolarization angles) as reconstructed in their wavelength dependence from a direct, *i.e.*, non-analytical, wavelength-per-wavelength inverse Kramers-Krönig transformation of the above complex refractive index. If the oscillator in question was the only one present in the optical band gap of $Zn_{0.74}Mg_{0.26}Se$, then it would probably be detected by ellipsometry. In reality, with $Zn_{0.74}Mg_{0.26}Se$ one faces a continuum of impurity levels by ellipsometry – apparent in the photoluminescence data (see main text), and not only the particular oscillator(s) selectively activated out of such continuum in a resonant Raman experiment. As such, the latter resonance(s) cannot emerge from the continuum in the ellipsometry data.

Altogether, our crude one-oscillator/one-adjustable-parameter description used to explain the local inversion of the refraction index dispersion near ω_i (Fig. 2a) – taken as responsible for the "tilt" of the Raman scan line (Fig. 1a), if not exact, remains useful to fix ideas.

4.4. Resonance-induced activation of the $\left(PP_{LO}^{int}, PP^{+}\right)$ Raman signals. As apparent in Fig. S10, which displays a series of extended near-forward Raman spectra taken with the red (a, R-6632.8 nm) and green (b, G-514.5 nm) laser lines at various scattering angles, the *sine qua non* condition for the observation of the $\left(PP_{LO}^{int}, PP_{LO}^{+}\right)$ Raman signals is to polarize the incident laser beam perpendicular to the $\vec{c}$ −crystal axis. Otherwise the resonance process behind the two modes is inhibited and they are not visible. In fact the $\left(PP_{LO}^{int}, PP_{LO}^{+}\right)$ are absent when $\vec{e}_i \parallel \vec{c}$ and present when $\vec{e}_i \perp \vec{c}$. Once activated ($\vec{e}_i \perp \vec{c}$) the resonance-induced Raman scattering is more or less pronounced depending on the used laser line, with concomitant impact on the $\left(PP_{LO}^{int}, PP_{LO}^{+}\right)$ Raman intensities. Therefore the laser pulsation (ω_i) is not as much restrictive as the laser polarization. This suffices to rule out any cavity effect[20] between the parallel crystal faces as the possible origin of the $\left(PP_{LO}^{int}, PP_{LO}^{+}\right)$ Raman signals.

In each (R, G) near-forward Raman series the localized impurity levels taken as responsible for the near-resonant activation of the PP_{LO}^{int} and PP_{LO}^{+} lines is identified through a dramatic enhancement of the Raman signal far away from ω_i. The corresponding pulsations of impurity levels are shifted by ~840 cm^{-1}, ~571 cm^{-1} and ~482 cm^{-1} beneath the R and G laser pulsations, respectively (recall the Stokes scattering). The enhanced Raman signal close to the latter pulsations is of the phonon-polariton type since it vanishes along with the $\left(PP_{LO}^{int}, PP_{LO}^{+}\right)$ modes when the scattering angle θ increases, whichever laser line is used. Most probably the enhanced signal is PP^{+} −like since the upward-tilted

Raman scan line addressed when using the $\vec{e}_i \perp \vec{c}$ polarization setup is nearly tangential to the PP^+ dispersion (Fig. 2b), so that the latter branch may provide a continuous range of candidates likely to be enhanced whichever impurity level is involved in the resonance process. As expected the $\left(PP_{LO}^{int}, PP_{LO}^{+}\right)$ features are best resolved (R vs. G) when the resonance is more pronounced, leading to a stronger enhancement of the Raman signal at the relevant resonance (marked with label "Res." at 840 cm^{-1} and 571 cm^{-1} below the pulsations of the R and G laser lines in Fig. S10, respectively).

Supplementary information – only references

1. Polian, A., Simon, P. & Pagès, O. Thermodynamic Properties of Solids – Experiment and Modeling (Chaplot, S. L., Mittal, R. & Choudhury, N. eds.). Chap. 2, 10 (Wiley-VCH, 2010).
2. Hennion, B., Moussa, F., Pépy, G. & Kunc, K. Normal modes of vibration in ZnSe. *Phys. Lett. A* 36, 376 – 378 (1971).
3. Tian, Z. *et al.* Inelastic X-ray scattering of phonon dispersion and lifetimes in $PbTe_{1-x}Se_x$ alloys. *J. Phys. Condens. Matter* 27, 375403-1 – 375403-5 (2015).
4. Ya Valakh, M. *et al.* Anharmonic coupling of phonon modes in $Zn_xCd_{1-x}Se$ crystals. *Phys. Stat. Sol.* (b) 113, 635 – 645 (1982).
5. Pradhan, G. K. *et al.* Pressure-induced phonon freezing in the $Zn_{1-x}Be_xSe$ alloy: A study via the percolation model. *Phys. Rev. B* 81, 115207-1 – 115207-6 (2010).
6. Firszt, F., Szatkowski, J. & Męczyńska, H. Luminescence and electrical properties of $Zn_{1-x}Mg_xSe$ mixed crystals grown by high pressure Bridgman method. *Electron. Technol*. 27, 37 – 47 (1994).
7. Paszkowicz, W., Spolnik, Z., Firszt, F. & Męczyńska, H. Powder diffraction study of $Zn_{1-x}Mg_xSe$ alloys. Proceedings of the 45th Annual Conf. on Applications of X-ray Analyses (Denver, Colorado, USA, August 1996), *Adv. X-ray Anal.* 40, 704 – 709 (1997).
8. Pandely, R., Lepak, P. & Jaffe, J. E. Electronic structure of alkaline-earth selenides. *Phys. Rev. B* 46, 4976 – 4977 (1992).
9. Huang, D. *et al.* Structural and optical properties of $Zn_{1-x}Mg_xSe$ alloys grown on GaAs(001) substrates by molecular beam epitaxy. *J. Cryst. Growth* 184/185, 1085 – 1089 (1998).
10. Paszkowicz, W., Dłużewski, P., Spolnik, Z. M., Firszt, F. & Męczyńska, H. Formation of 4H and 8H polytypes in bulk $Zn_{1-x}Mg_xSe$ crystals. *Journal of Alloys and Compounds* 286, 224 – 235 (1999).
11. Bhalerao, G. M. *et al.* High-pressure X-ray diffraction and extended X-ray absorption fine structure studies of ternary alloy $Zn_{1-x}Be_xSe$. *J. Appl. Phys.* 108, 083533-1 – 083533-7 (2010).
12. Zagoruiko, Y. A., Fedorenko, O. A., Kovalenko, N. O., Rom, M. A. & Mateychenko, P. V. Physical properties of ZnSe-MgSe, ZnSe-CdSe solid solutions and possibilities of their application in IR engineering. *Semiconductor Physics – Quantum Electronics & Optoelectronics* 3, 165 – 169 (2000).
13. Puzikov, V. M., Zagoruiko, Y. A., Fedorenko, O. A. & Kovalenko, N. O. Optical and electro-optical properties of ZnMgSe single crystals. *Crystallography Reports* 49, 215 – 216 (2004).
14. Poulet, H. & Mathieu, J. –P. Spectres de Vibration et Symétrie des Cristaux. Chap. 9, 244. (Gordon and Breach, 1970).
15. Brafman, O. Phonons and structure of $Zn_xCd_{1-x}Se$ solid solutions. *Solid State Commun.* 11, 447 – 451 (1972).
16. Vérié, C. Beryllium substitution-mediated covalency engineering of II-VI alloys for lattice elastic rigidity reinforcement. *J. Cryst. Growth* 184/185, 1061 – 1066 (1998).
17. Soler, J. M. *et al.* The SIESTA method for *ab initio* order-N materials simulation. *J. Phys.: Condens. Matter* 14, 2745 – 2779 (2002).
18. Kunc, K. & Martin, R. M. Density-functional calculation of static and dynamic properties of GaAs. *Phys. Rev. B* 24, 2311 – 2314 (1981).
19. Troullier, N. & Martins, J. L. Efficient pseudopotentials for plane-wave calculations. *Phys. Rev. B* 43, 1993 – 2006 (1991).
20. Vinogradov, E. A. Semiconductor microcavity polaritons. *Physics – Uspekhi* 45, 1213 – 1250 (2002).

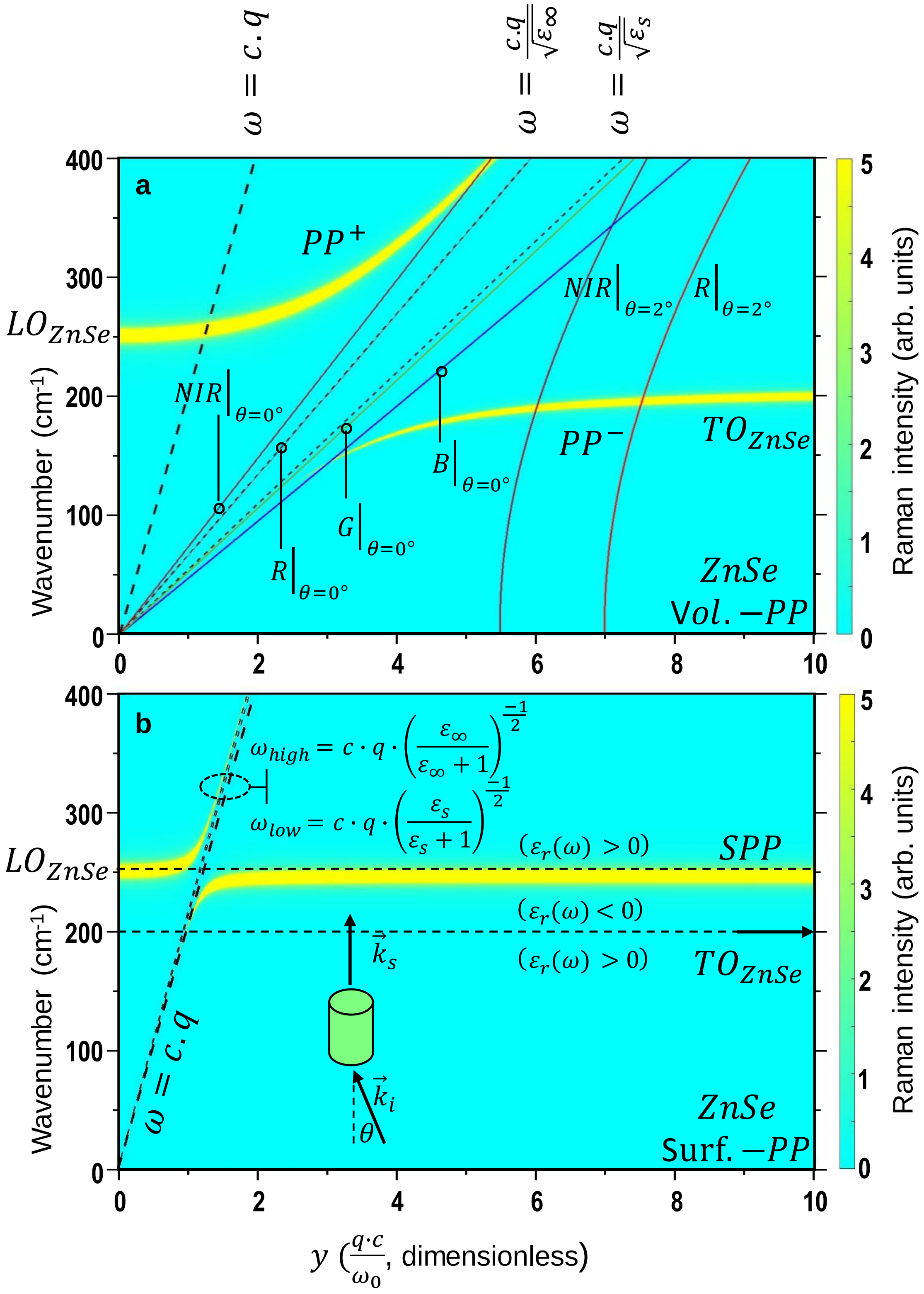

Figure S1 | Reference *VPP* and *SPP* dispersions of ZnSe. Theoretical dispersions of the volume (**a**, VPP) and surface (**b**, SPP) phonon-polaritons (PP) of ZnSe with zincblende structure, propagating outside $[\varepsilon_r(\omega) > 0]$ and inside $[\varepsilon_r(\omega) < 0]$ the *Reststrahlen* $TO - LO$ band, respectively. The relevant photon-like asymptotes are specified in each case (dotted lines). The 'scan' lines achieved in a Raman scattering experiment done in the perfect forward scattering geometry ($\theta = 0°$) with the NIR (785.0 nm), R (632.8 nm), G (514.5 nm) and B (488.0 nm) laser lines, calculated using the dispersion of the refractive index taken from Ref. 37 (main text), are indicated. As small scattering angle as 2° suffices to recover the asymptotic purely-mechanical regime away from Γ (y=0).

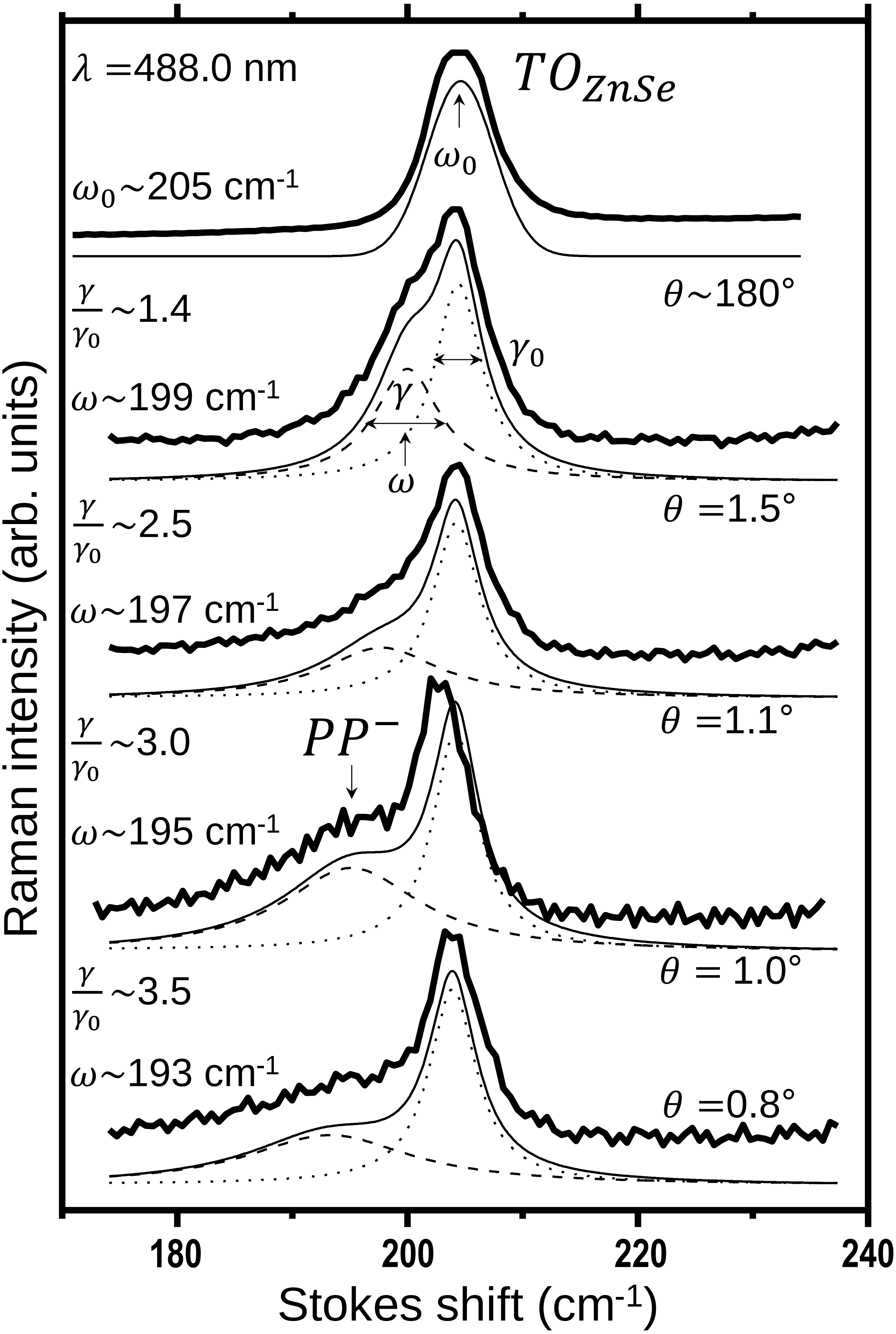

Figure S2 | Reference near-forward Raman study of ZnSe. Near-forward Raman spectra taken with the zincblende-type ZnSe compound using the blue (488.0 nm) laser line. The damping of the PP^- mode (γ) progressively enlarges by penetrating deeper into the phonon-polariton regime, as indicated. The scattering angle (θ) for each spectrum is estimated based on identification of the relevant scan line addressing the pulsation (ω) of the detected PP^- feature (see Fig. S1).

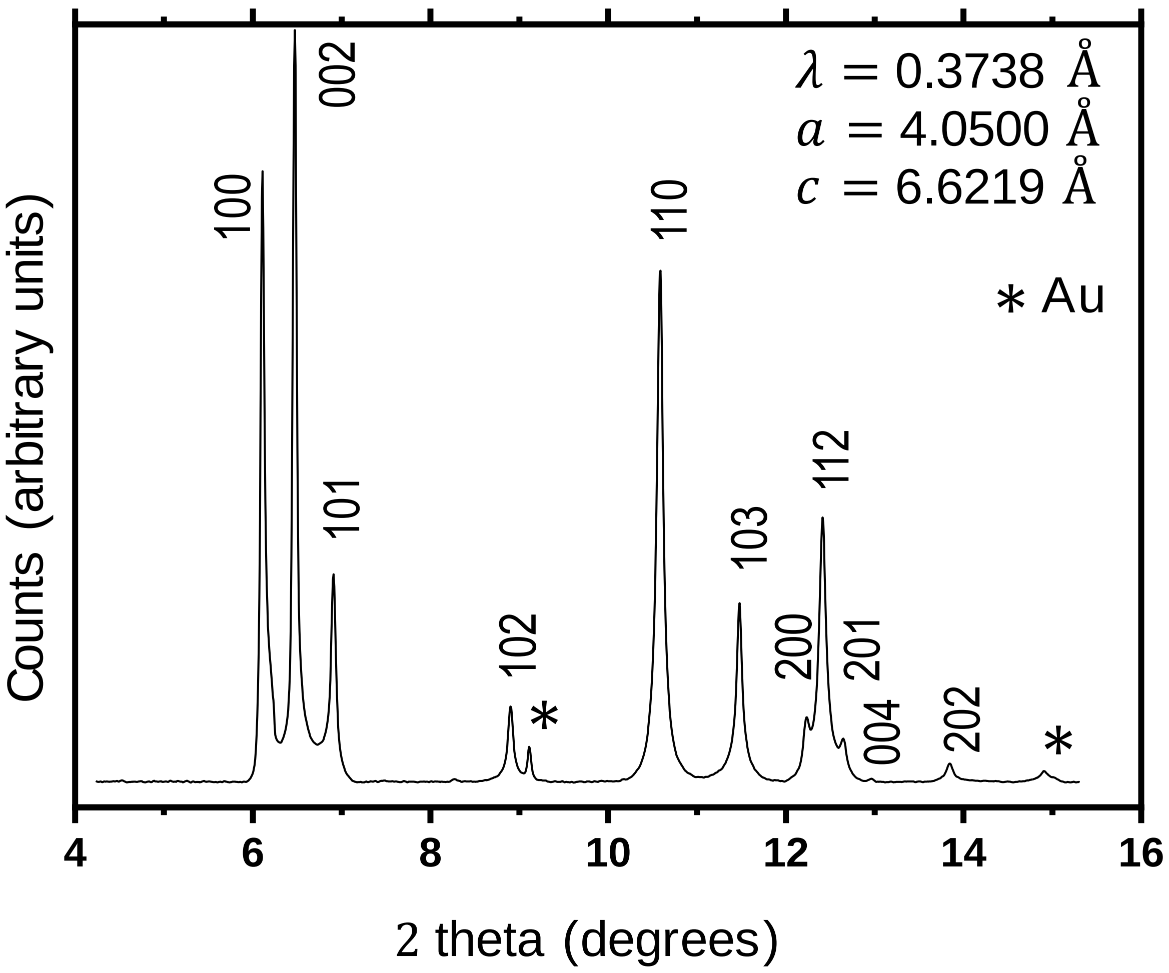

Figure S3 │ X-ray diffractogram. Powder X-ray diffractogram obtained at ambient pressure with the $Zn_{0.74}Mg_{0.26}Se$ mixed crystal using the 0.3738 Å radiation of the PSICHÉ beamline at the SOLEIL synchrotron. The corresponding a and c lattice constants, from which the composition of the mixed crystal was derived (based on Ref. 31 – main text), are indicated. The asterisk marks diffraction lines due to Au, used for pressure calibration.

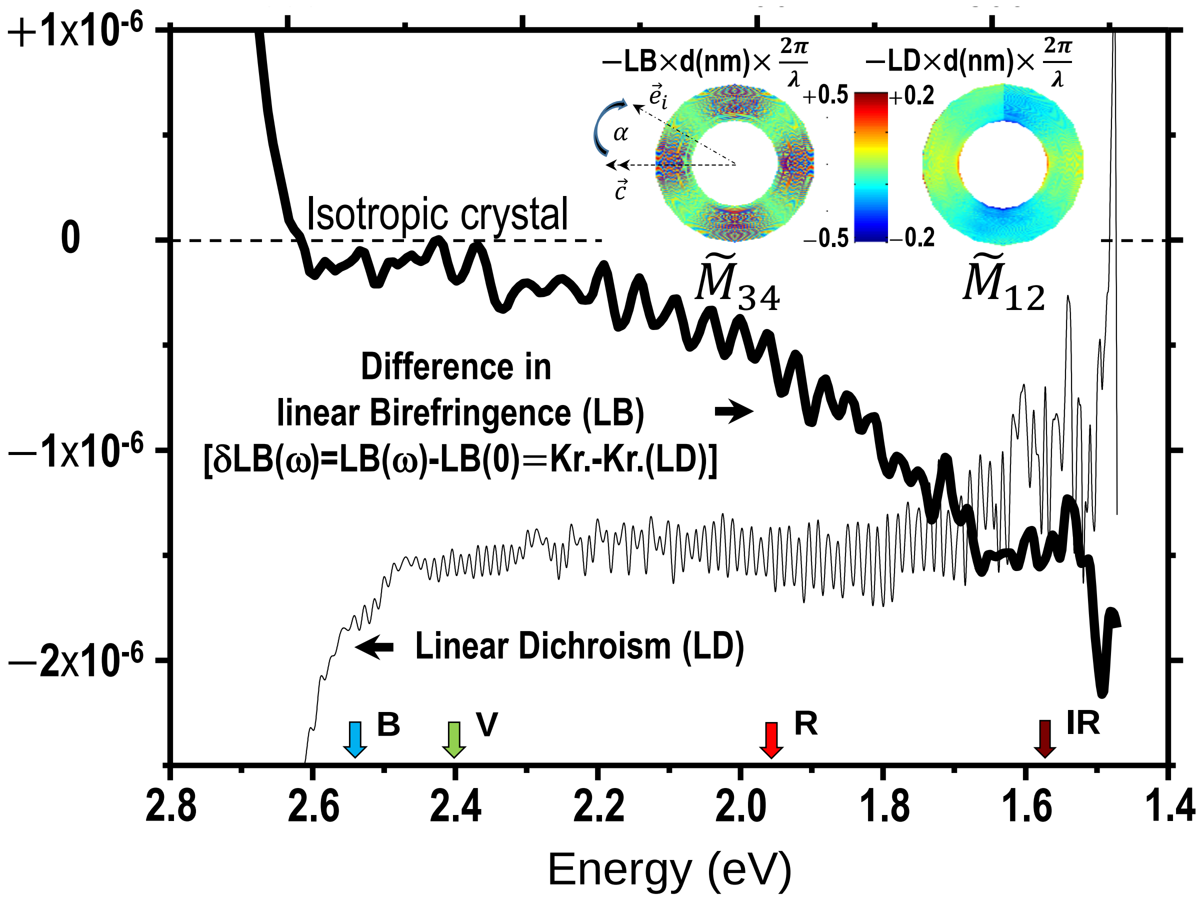

Figure S4 | Ellipsometry measurements. Transmission ellipsometry measurements done in the visible spectral range at normal incidence throughout the parallel crystals faces with in-plane $\vec{c}$ −axis (at distance d from each other) of the oriented $Zn_{0.74}Mg_{0.26}Se$ crystal. The corresponding $\widetilde{M}_{12}$ and $\widetilde{M}_{34}$ elements of the Müller's matrix, related to the linear dichroism and to the linear birefringence, respectively, are shown by adopting a circular representation and a color code. Each circle captures the spectral dependence (along the radius – the wavelength λ increase from inside to outside) as well as the dependence on the incident polarization ($\vec{e}_i$) rotated by the angle α from the $\vec{c}$ −crystal axis, as indicated. The linear dichroism (thin curve) measured along the $\vec{c}$ −axis ($\alpha = 0°$) throughout the visible spectral range is plotted. An access to the difference in linear birefringence $LB(\omega) - LB(0)$ (thick curve) is achieved by applying the Kramers-Krönig transformation to the raw data of linear dichroism.

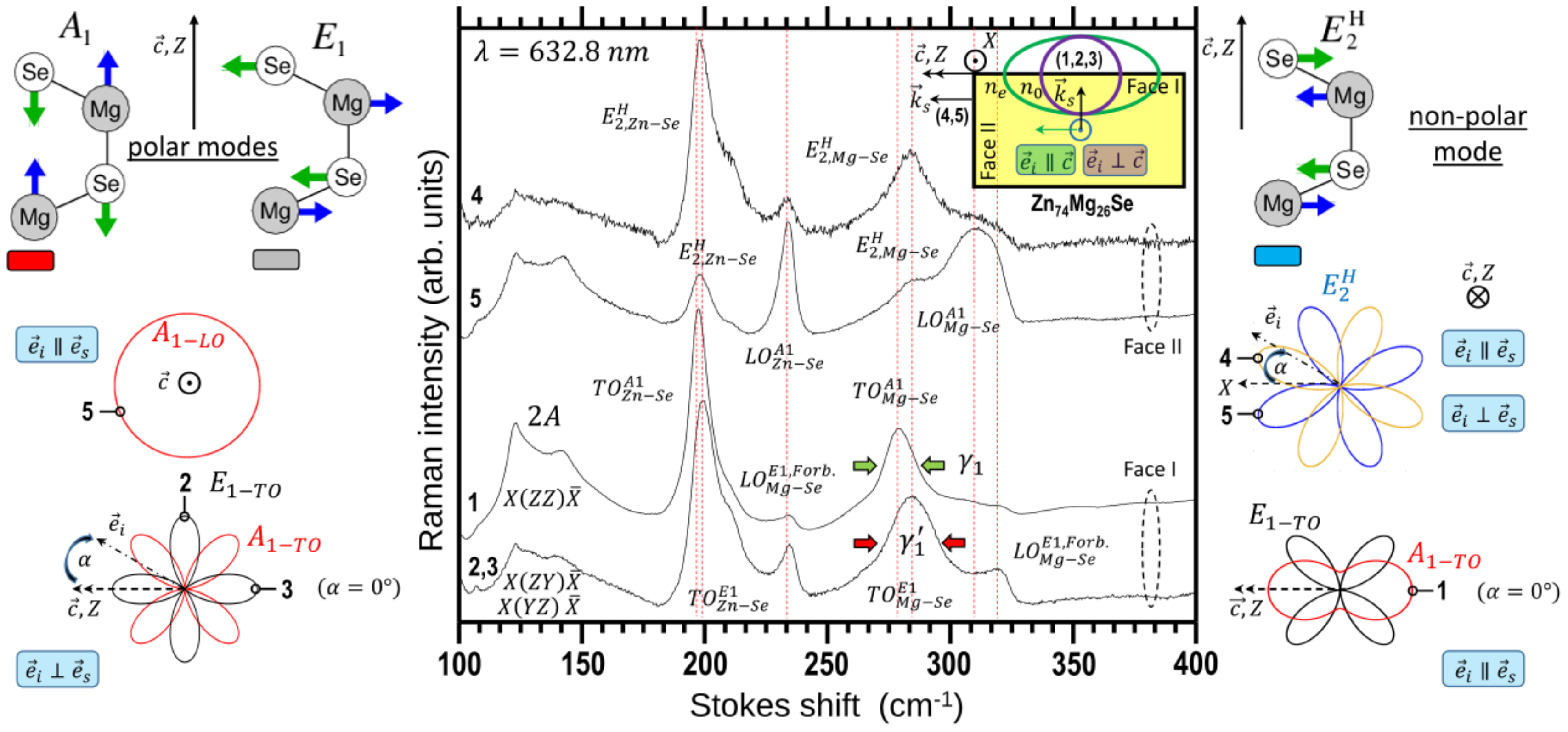

Figure S5 │ Reference Backscattering Raman spectra of the native TO modes behind the VPP's. Polarized Raman spectra in the various backscattering geometries in reference to the $((X, Y, Z)$ −laboratory axis. The dependence of the Raman intensity on the angle (α) between the incident polarization $(\vec{e}_i)$ and the $(\vec{c}, Z)$ −axis (face I) or the in-plane X −axis perpendicular to $\vec{c}$ (face II) when using parallel $(\vec{e}_i \parallel \vec{e}_s)$ and crossed $(\vec{e}_i \perp \vec{e}_s)$ polarizations are indicated (multi-lobe patterns) for each relevant phonon symmetry, *i.e.*, the polar (A_1, E_1) as well as non-polar (E_2^H) ones, using a color code. The corresponding vibrations are sketched out in each case. In particular, spectra 1 and 2 provide reference insights into the native A_1 and E_1 TO modes behind the VPP's.

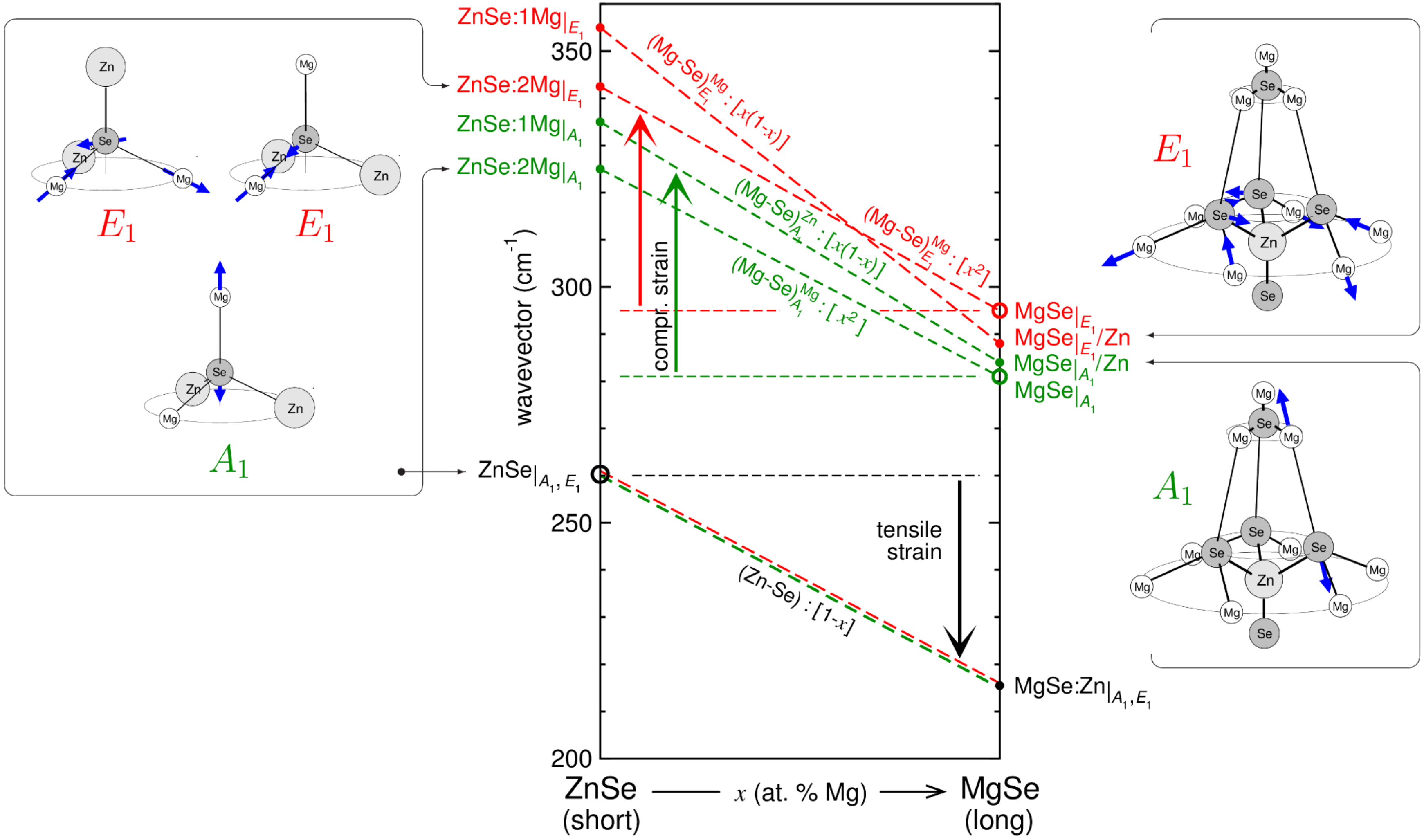

Figure S6 │ ***Ab initio*** **insight into the wurtzite-type percolation TO pattern of $Zn_{1\text{-}x}Mg_xSe$.** Percolation-type A_1 and E_1 purely-mechanical TO modes (dashed lines) of the wurtzite-type $Zn_{1\text{-}x}Mg_xSe$ mixed crystal, derived from *ab initio* phonon calculations done with prototypal Zn- and Mg-based impurity motifs in the (Zn,Mg)-dilute limits (x~0,1). The relevant vibrational patterns are shown. The position of the impurity modes (plain symbols) with respect to the corresponding parent modes (empty symbols) is governed by the local strain, as indicated.

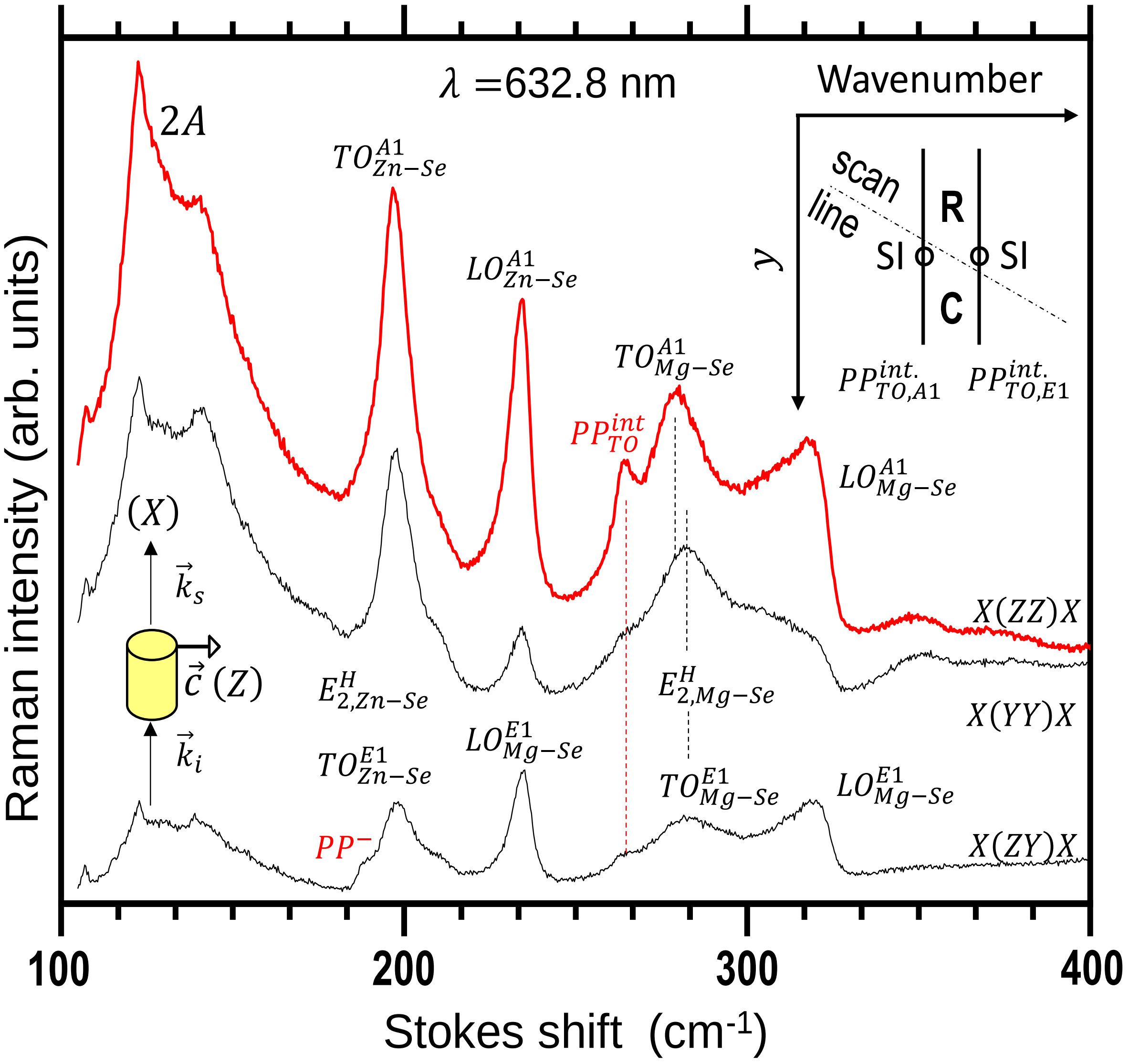

Figure S7 │ Near-forward-scattering Raman spectra of $Zn_{0.74}Mg_{0.26}Se$. Polarized near-perfect forward Raman spectra ($\theta \sim 0°$) taken with the red laser line in the $X(YY)X$ and $X(ZY)X$ scattering geometries. The corresponding $X(ZZ)X$ spectrum is added for comparison. A diagram illustrates how the Raman scan line crosses the A_1-like and E_1-like PP^{int} dispersions on each side of the singularity (SI, standing for I_c), corresponding to different access to the PP^{int} mode in the $X(ZZ)X$ and $X(YY)X$ geometries, *i.e.*, in the reinforcement (R) and collapse (S) regimes, respectively. The modes are labelled using the same contour/area code as in Fig. S5, for a direct correspondence.

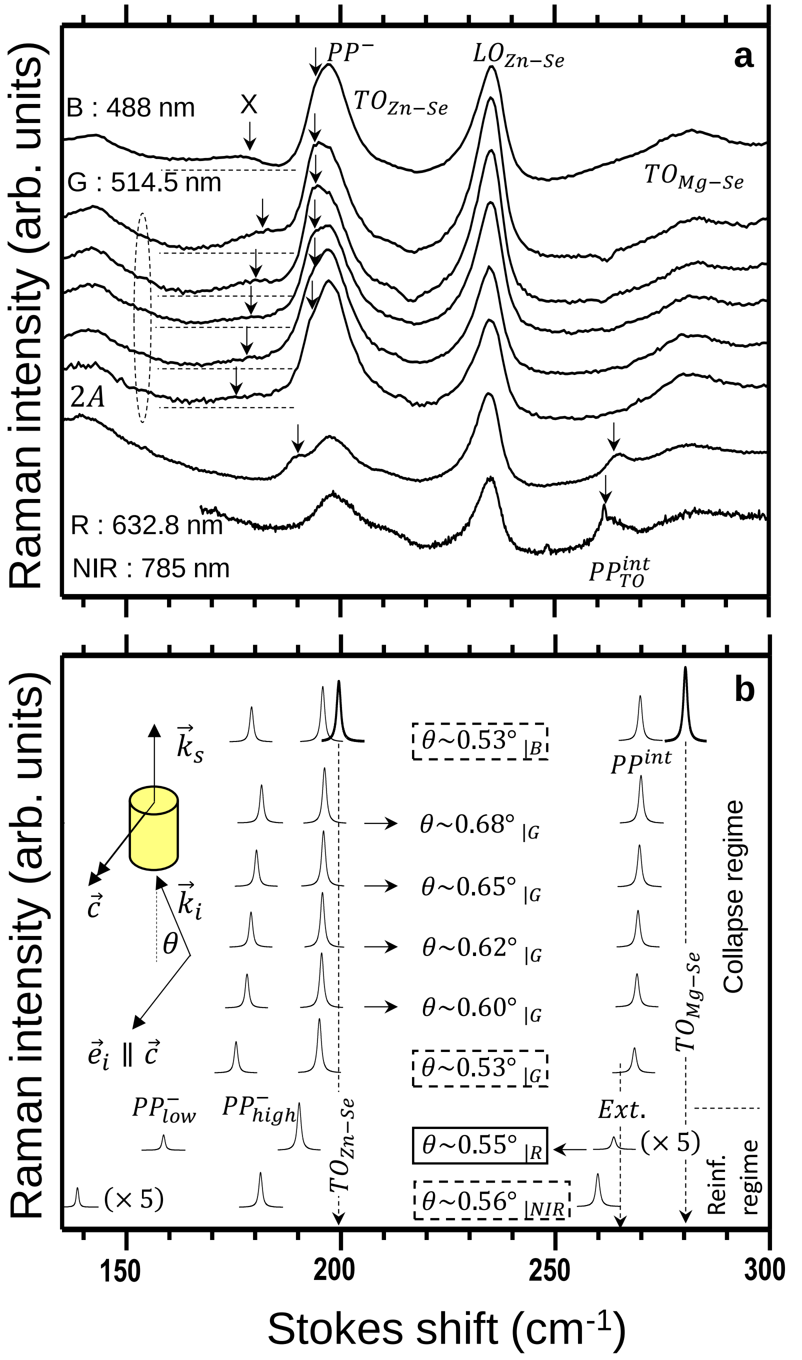

Figure S8 │ Combined contour modeling of the near-forward $(PP_{low}^-, PP_{high}^-)$ Raman signals. a Unanalyzed near-forward (θ~0°) $Zn_{0.74}Mg_{0.26}Se$ Raman spectra recorded by using various laser lines (near-infrared-NIR, red-R, green-G, blue-B) with the incident polarization ($\vec{e}_i$) parallel to the $\vec{c}$ −crystal axis. **b** Fair contour modeling of the phonon-polariton Raman $(PP_{low}^-, PP_{high}^-, PP_{TO}^{int})$ lineshapes in their dependence on both the scattering angle (θ) and the laser line is achieved by awarding 72% and 12% of the available ZnSe-like oscillator strength to the 'fractional' PP_{low}^- and PP_{high}^- modes, respectively. The reference scattering angle for the red laser line (solid frame) was estimated from the pulsation of the PP_{TO}^{int} mode. The corresponding scattering angles for the (NIR,G,B) laser lines (dotted frames), obtained via the dispersion of the effective refractive index measured by ellipsometry, are used to estimate the fractions of ZnSe-like oscillator strength awarded to the 'fractional' PP_{low}^- and PP_{high}^- modes. Alternative (unframed) scattering angles for the G laser line are estimated from the pulsations of the latter two modes.

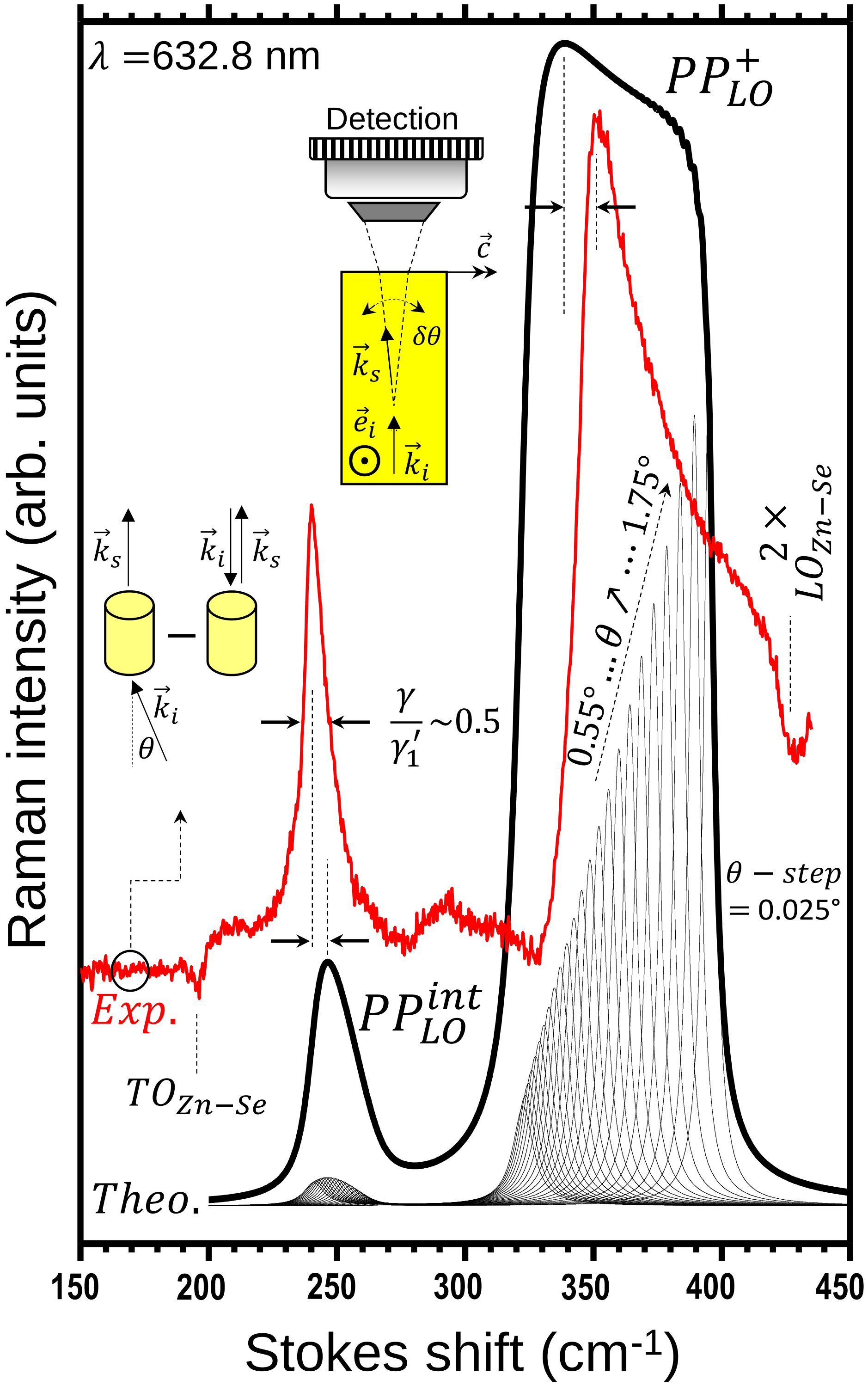

Figure S9 │ Combined contour modeling of the near-forward $(PP_{LO}^{int}, PP_{LO}^{+})$ Raman signals. Contour modeling of the $Zn_{0.74}Mg_{0.26}Se$ near-forward $(\theta \sim 0°)$ PP_{LO}^{int} and PP_{LO}^{+} Raman signals (smooth-thick curve) obtained with the red (632.8 nm) laser line polarized perpendicular to the $\vec{c}$ −crystal axis. A distribution in scattering angle $[0.55°, 1.75°]$ is used to take into account the finite numerical apertures of the lens used to collect the scattered light The elementary $(PP_{LO}^{int}, PP_{LO}^{+})$-joint Raman lineshapes calculated each step increase of 0.025° in the scattering angle are shown underneath the main (envelope) curve resulting from their sum. The corresponding experimental Raman signal (noisy-thin curve), obtained by subtraction of the backscattering-like Raman spectrum (Fig. 2b, bottom curve) to the near-perfect forward Raman signal (Fig. 1a, curve 2), is shown for comparison.

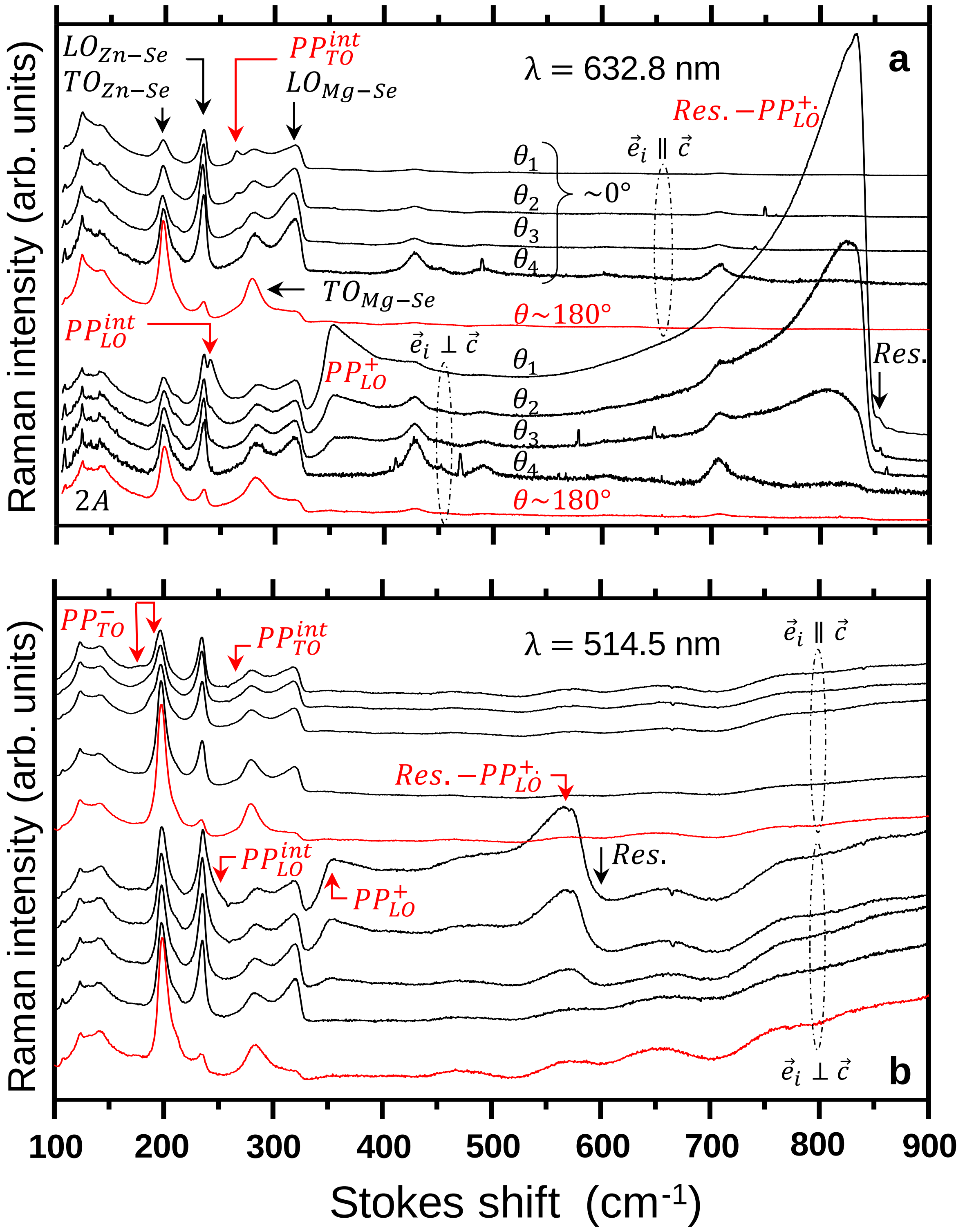

Figure S10 │ **Laser–dependence of the near-forward Raman spectra.** Extended near-forward $Zn_{0.74}Mg_{0.26}Se$ Raman spectra taken at the same sample spot, using the same external incidence and detection angles probing the deep (from top to bottom) phonon-polariton regimes ($\theta \leq 2°$), with the red (**a**, 632.8 nm, selection from Fig. 2b) and green (**b**, 514.5 nm) laser lines, by polarizing the incident laser beam parallel ($\vec{e}_i \parallel \vec{c}$) or perpendicular ($\vec{e}_i \perp \vec{c}$) to the $\vec{c}$ −crystal axis. The scattered light is not analyzed. The phonon-polariton features in each geometry are indicated using clear symbols, together with local resonances involving impurity levels ($Res.$). The backscattering Raman spectra taken at the same sample spot in the same conditions are added (clear curves) for reference purpose.
